# Predictive Screening of Ta_4_C_3_ MXene as an Inhalable Nanotherapeutic Based on an Advanced 3D Air–Liquid Interface Lung Model

**DOI:** 10.1021/acsnano.6c03602

**Published:** 2026-04-02

**Authors:** Ying Kong, Nicole J. Machi, Fuze Jiang, Eszter J. Barthazy Meier, Viktoria Agarwal, Zhou Dong, Sung Sik Lee, Jing Wang

**Affiliations:** † Institute of Environmental Engineering, ETH Zürich, 8093 Zürich, Switzerland; ‡ Scientific Center for Optical and Electron Microscopy (ScopeM), ETH Zürich, 8093 Zürich, Switzerland; § Institute of Biochemistry, ETH Zürich, 8093 Zürich, Switzerland; ∥ Department of Information Technology and Electrical Engineering, ETH Zürich, 8092 Zürich, Switzerland; ⊥ Department of Health Sciences and Technology, ETH Zürich, 8092 Zürich, Switzerland; # Laboratory for Building Energy Materials and Components, Empa, Swiss Federal Laboratories for Materials Science and Technology, 8600 Zürich, Switzerland

**Keywords:** inhalable nanotherapeutic, 3D ALI lung model, Ta_4_C_3_ MXene, anti-inflammatory, antifibrotic, antioxidant, predictive screening

## Abstract

The rapid development of two-dimensional (2D) MXenes has outpaced our understanding of their pulmonary safety, leaving a critical gap in clinical translation due to inconsistent data from traditional 2D cell cultures. Herein, we developed an immunocompetent three-dimensional (3D) alveolar model comprising A549 epithelial cells, MRC-5 fibroblasts, and THP-1-derived macrophages cultured at the air–liquid interface. This self-organized triculture forms a stratified epithelial–mesenchymal trophic unit with functional surfactant production and cell–cell crosstalk, providing a physiologically relevant platform for the predictive screening of potential nanomedicines. Following thorough characterization, we utilized this system to investigate the therapeutic potential of in-house synthesized Ta_4_C_3_ MXene nanosheets across three size fractions (100–500 nm, 500–2000 nm, and ≥2000 nm). Key biological events leading to lung inflammation and fibrosis, including reactive oxygen species (ROS) accumulation and the release of pro-inflammatory and pro-fibrotic markers, demonstrated the responsiveness of the model. All of the size fractions showed high biocompatibility. Cryogenic transmission electron microscopy and energy-dispersive X-ray spectroscopy confirmed efficient cellular internalization. Notably, the 100–500 nm fraction induced the most pronounced therapeutic reaction by scavenging ROS and promoting macrophage polarization shift from M1 to M2 and arresting fibrotic remodeling. The addition of macrophages in the tricultures led to heightened inflammatory and fibrotic responses, enabling more sensitive detection of the anti-inflammatory and antifibrotic effects of Ta_4_C_3_ MXenes. This study establishes a rapid 3D alveolar model for predictive assessment of pulmonary safety and therapeutic efficacy upon Ta_4_C_3_ treatment.

## Graphical abstract

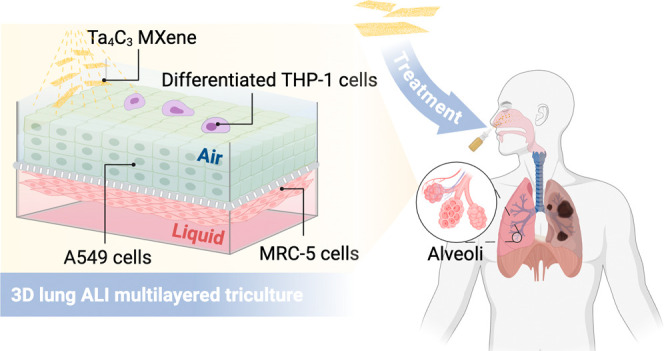

Pulmonary diseases, such as acute lung injury and fibrosis, represent a significant global health burden, necessitating innovative therapeutic strategies that can directly target the affected tissue with minimal systemic exposure. Inhalation-based drug administration offers a noninvasive approach for treating pulmonary diseases by delivering therapeutics directly to the lungs while reducing systemic side effects.^1–4^ While various nanomaterials are being explored for this purpose, an innovative class of two-dimensional (2D) materials known as MXenes—with prominent examples including titanium carbide variants, has garnered significant attention for a range of biomedical applications.^5–7^ Based on their unique physicochemical and biological properties, MXenes stand out as theoretically compelling candidates for development into inhalable therapeutics; however, this specific avenue remains largely unexplored. These materials possess a unique combination of advantageous surface chemistry, high surface area, and intrinsic multifunctional properties, including potent antioxidant,^8–10^ anti-inflammatory,^11,12^ and tissue-regenerative effects,^13^ which are highly relevant for combating complex lung pathologies. Notably, MXene synthesis can yield particles across a broad size spectrum (often ranging from ∼100 nm to several micrometers), and it is well-established that particles with aerodynamic diameters below approximately 2 μm can efficiently penetrate the deep lung to reach the alveolar region.^14^ This amenability to aerosolization and potential for targeted alveolar delivery, coupled with their intrinsic therapeutic properties, strongly suggests their promise as locally acting inhalation drugs.^15^ However, despite these promising attributes, the dedicated exploration and preclinical screening of MXenes as specifically formulated inhalable medicines remain largely uncharted. Critical gaps persist in understanding how MXene characteristics influence their interactions with the intricate lung epithelial environment and their therapeutic efficacy following pulmonary delivery.^16^

Translating the potential of any novel material into a viable clinical treatment requires a robust preclinical evaluation. Traditional reliance on animal testing is often time-consuming, costly, and exhibits limitations in accurately predicting human physiological and pathological responses,^17^ prompting a legislative shift toward new alternative methods (NAMs).^18^ Microphysiological systems (MPS), which replicate lung tissue functions in laboratory settings, have emerged as safer and more physiologically accurate tools for advancing inhalation drug development.^19^ While conventional 2D submerged lung cell cultures have been employed, they fail to replicate crucial aspects of native lung tissue, particularly the air–epithelium interaction critical for studying inhaled substances.^20,21^ Consequently, three-dimensional (3D) MPS, especially those incorporating an air–liquid interface (ALI), have gained prominence.^22^ ALI systems expose lung epithelial cells apically to air, fostering physiologically relevant cellular differentiation, barrier formation, and cell–cell interactions.^23,24^ This advanced culture environment enables more realistic drug administration protocols and provides an invaluable platform for rigorously investigating the localized effects of potential inhalable therapeutics like MXenes.^25^

To bridge the translational gap between nanomaterial synthesis and pulmonary safety, the present study details the development of a sophisticated immunocompetent 3D ALI triculture (Figure 1). As illustrated, this model integrates A549 epithelial cells, MRC-5 fibroblasts, and THP-1-derived macrophages to reconstitute the epithelial–mesenchymal trophic unit (EMTU) in vitro. Utilizing this physiologically representative platform, we tracked the complete path of in-house synthesized Ta_4_C_3_ MXene nanosheets—from chemical exfoliation and size fractionation to cellular internalization and subsequent lysosomal sequestration. We performed a comprehensive safety-to-efficacy evaluation, beginning with cytotoxicity monitoring to establish biocompatibility thresholds across all size fractions (100 nm, 500–2000 nm, and ≥2000 nm). This was followed by a systematic assessment of how lateral dimensions influence the ability of Ta_4_C_3_ to scavenge ROS, modulate macrophage polarization, and arrest TGF-β-mediated fibrotic remodeling. This work aims to map the precise structure–activity relationships governing MXene pulmonary safety and therapeutic efficacy, demonstrating the utility of advanced 3D models in establishing the design rules for next-generation inhalable nanomedicines. Such a comprehensive approach provides critical insights into MXene-lung interactions and establishes a robust foundation for future preclinical studies.

**Figure 1:**
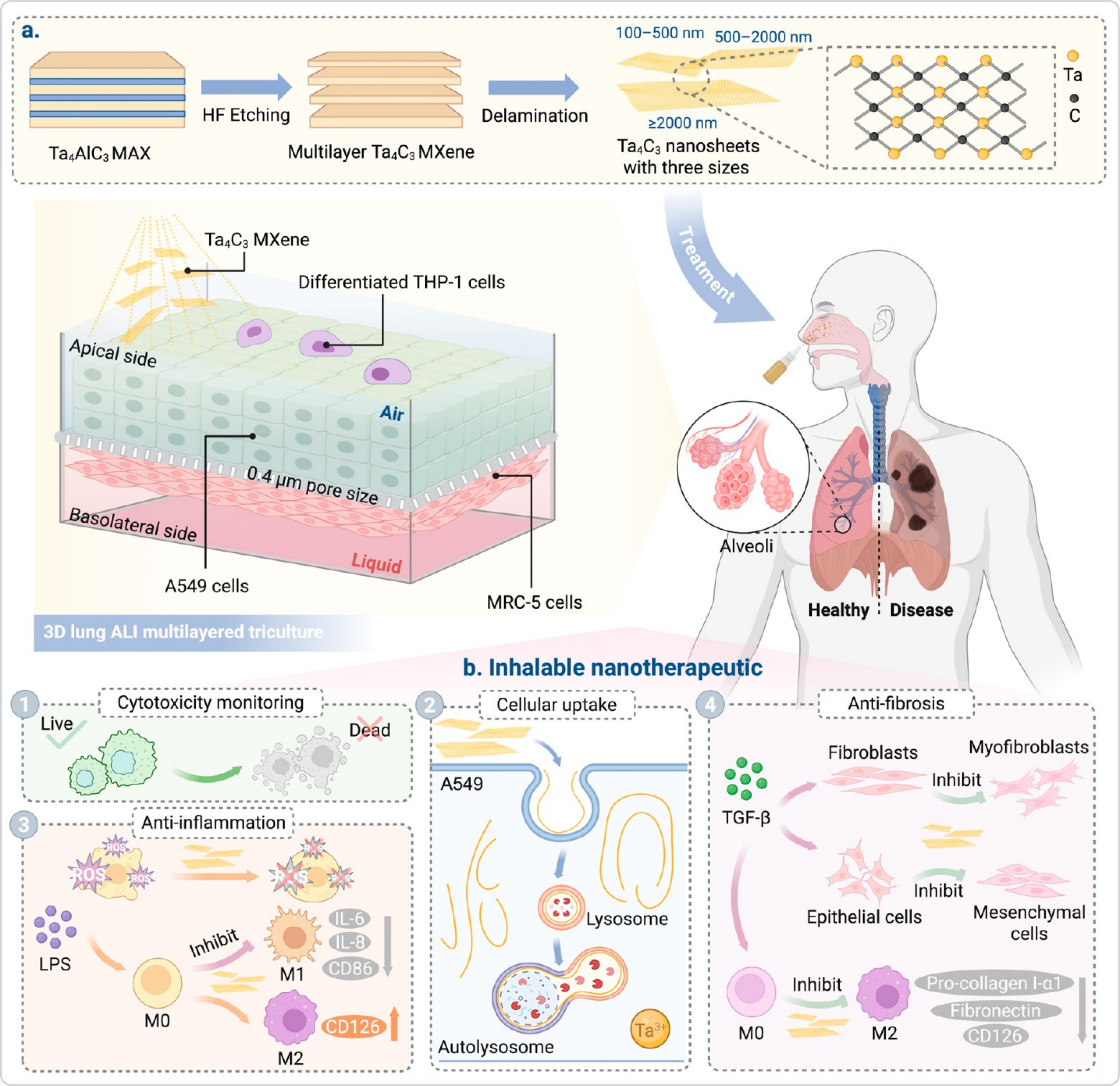
Evaluation of size-tunable Ta_4_C_3_ MXene as an inhalable nanotherapeutic in a 3D ALI lung triculture model. (a) Top-down synthesis of Ta_4_C_3_ MXene nanosheets via selective HF etching of Ta_4_AlC_3_ and delamination into three distinct lateral size fractions (100–500 nm, 500–2000 nm, ≥2000 nm) to assess size-dependent respiratory therapeutic efficacy. The schematic outlines the inhalable delivery of these nanosheets to a 3D lung ALI triculture system. This model incorporates differentiated THP-1 macrophages (apical), A549 epithelial cells (apical), and MRC-5 fibroblasts (basolateral) on a Transwell (0.4 μm porous size) membrane. (b) Mechanistic pathways of the inhalable Ta_4_C_3_ nanomedicine as a targeted therapeutic treatment for pulmonary disorders. (1) Size and dose-dependent cytotoxicity. (2) Cellular uptake and intracellular trafficking in A549 cells. (3) Anti-inflammatory activity; Ta_4_C_3_ nanosheets provide localized ROS scavenging and inhibit LPS-induced M1 macrophage polarization, reducing pro-inflammatory cytokines (IL-6, IL-8), and promoting the M2 phenotype. (4) Antifibrotic efficacy; Ta_4_C_3_ nanosheets arrest TGF-β-induced fibroblast-to-myofibroblast transition (FMT) and epithelial-to-mesenchymal transition (EMT) and suppress the pro-fibrotic M2 phenotype by downregulation of CD126, pro-collagen I-α1, and fibronectin.

## Results

### Formation and Characterization of 3D ALI Multilayered Tricultures

We set up the 3D triculture model on microporous Transwell inserts to mimic the alveolar environment. Within this scaffold, the cells self-organized into a clear stratified architecture. The MRC-5 fibroblasts on the basolateral side spanned the entire surface and had a flat, stretched-out shape. On the apical side, the A549 cells appeared more cuboidal and formed a solid epithelial sheet. To introduce immune competence, we seeded differentiated THP-1-derived macrophages onto the matured epithelium. As visualized in Figure 2a, these cells anchored to the apical surface, completing the triculture architecture. Once integrated, the macrophages organized into clusters clearly distinguishable by CD11b staining (Figure 2b), providing frontline defense and the necessary epithelial-immune crosstalk for our subsequent pathological challenges. Notably, while the native human alveolar wall is typically characterized by a single, ultrathin epithelial layer to facilitate gas exchange,^26^ the A549 cells in our model stacked up slightly to form 2–3 layers. This stratification created a robust epithelial–mesenchymal trophic unit (EMTU).^27,28^ It established a vital contact zone where epithelial cells and fibroblasts could physically interact and communicate, a structural feature often absent in standard separated tricultures. Once shifted to ALI conditions, this organization proved to be remarkably stable. Over 14 days of air-lift culture, the distinct cytoskeletal patterns—cortical F-actin in the epithelium and stress fibers in the fibroblasts—persisted even as cell density increased. Functionally, the epithelium underwent a significant maturation. As the air-lift phase progressed, the A549 cells produced increasing levels of pro-surfactant protein C (pSP-C), confirming their differentiation toward a functional Type II alveolar phenotype (Figure 2c–e).

**Figure 2:**
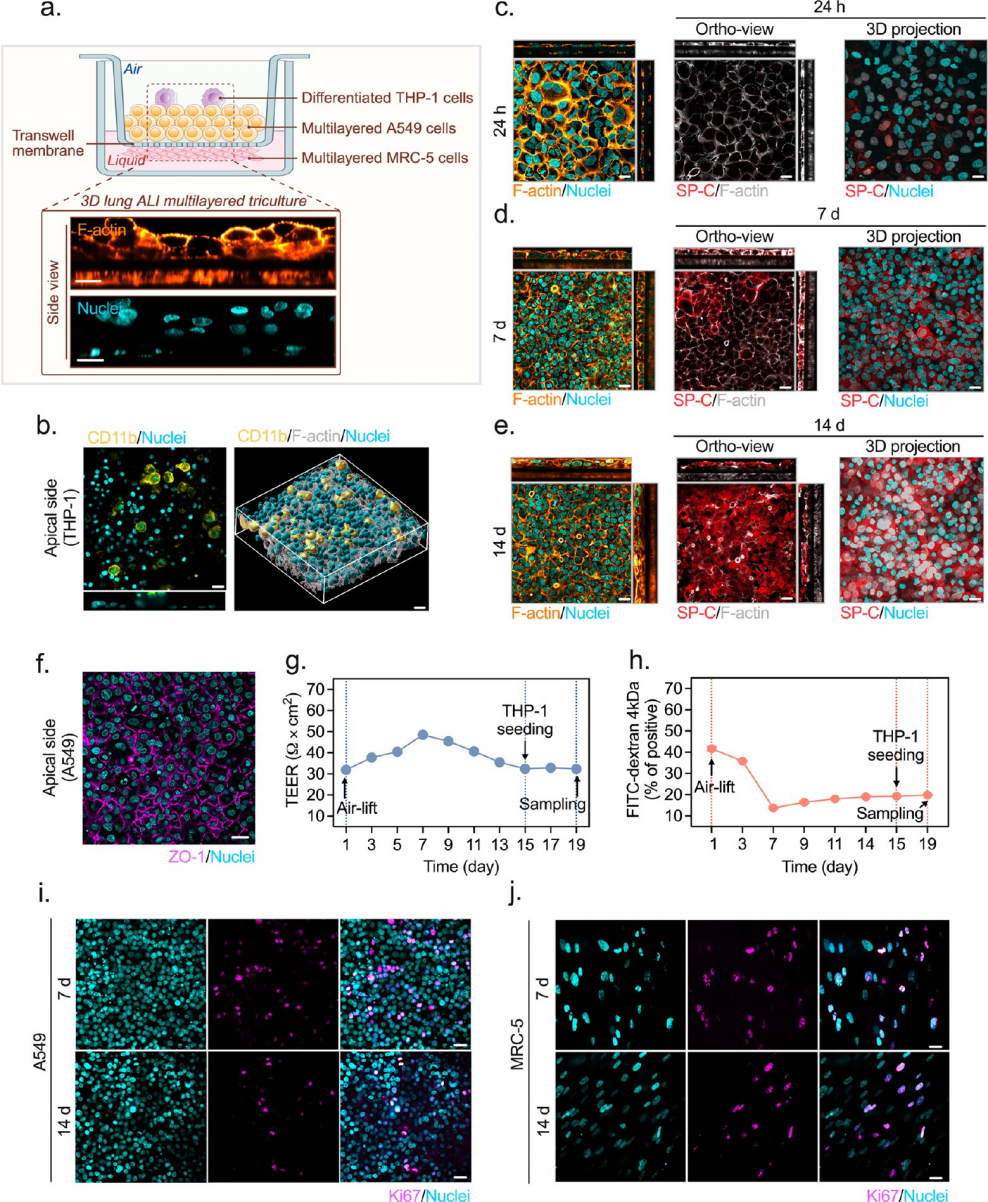
Structure and properties of the physiologically representative 3D lung ALI multilayered tricultures. (a) Schematic of the triculture architecture on a Transwell insert featuring differentiated THP-1 macrophages (apical), A549 epithelial cells (apical), and MRC-5 fibroblasts (basolateral). Representative spinning disk confocal microscopy (SDCM) side view displays cytoskeleton (F-actin, orange) and nuclei (cyan) organization at day 7. (b) Apical SDCM and 3D reconstructed views of CD11b-positive macrophages (yellow) on the top of epithelial layers. (c–e) Spatiotemporal evolution of the triculture over 14 days (24 h, 7 d, 14 d), showing cytoskeleton (F-actin, orange/gray) and alveolar type II marker pro-surfactant protein C (pSP-C, red) organization. (f) Immunofluorescence of the tight junction protein ZO-1 (magenta) at day 7. (g) Transepithelial electrical resistance (TEER) profiles monitored over 19 days. (h) Longitudinal paracellular permeability assessment via FITC-dextran (4 kDa) flux. Data in (g, h) indicate air-lift (day 1), THP-1 seeding (day 15), and sampling (day 19). Values represent mean ± SD (*n* = 10 for TEER; *n* = 3 for flux). (i, j) Proliferation kinetics (Ki67, magenta) of apical A549 cells and basolateral MRC-5 fibroblasts visualized at day 7 and day 14. Scale bars: 20 μm.

Since A549 cells alone failed to develop a tight, cohesive layer, we evaluated the integrity of the 3D triculture after the transition to ALI. The barrier matured rapidly following the day 1 shift. Transepithelial electrical resistance (TEER) climbed to a peak of 48.47 ± 0.20 Ω cm^2^ by day 7, while 4 kDa FITC-dextran leakage dropped to stable 15–20% baseline (Figure 2g,h). The resistance settled at approximately 30 Ω cm^2^ through THP-1 seeding (day 15) and sampling (day 19), and this maturation was mirrored by ZO-1 staining, which showed a continuous honeycomb-like network outlining apical cell boundaries (Figure 2f). The triculture consistently outperformed the A549 monoculture, which reached a peak of only 33.98 ± 0.50 Ω cm^2^ (Supporting Information Figure 1), a result in concordance with published data for this cell line.^29,30^ The data show that the interaction between epithelial cells and fibroblasts is necessary to form a robust, functional lung barrier.

To access the long-term stability of the model, we tracked the Ki67 expression in both A549 and MRC-5 cell populations over 14 days. While Ki67-positive cells were present throughout the culture period, the levels in the MRC-5 fibroblasts remained consistently lower than those in the A549 epithelium. This slower pace likely reflects the nonmalignant nature of fibroblasts and longer doubling time. As incubation time increased, the percentage of proliferating cells dropped in both layers (Figure 2i,j). This shift suggests that the cells transitioned into a quiescent, noncycling G0 state, a known behavior for long-term A549 cultures where high cell density modulates cell cycle genes.^31^ This downregulation of Ki67 indicates that the stratified triculture matured into a stable, resting tissue structure as the layers thickened.

### Prescreening of Diverse MXenes

Research into MXene-induced inflammatory responses has yielded inconsistent results,^32,33^ likely due to variations in chemical composition, particle size, and surface chemistry, alongside differing environmental contexts and cell types. To take the material properties into careful consideration within a pulmonary context, we conducted a systematic evaluation of five common MXenes, Ti_3_C_2_, V_2_C, Nb_2_C, Mo_2_C, and Ta_4_C_3_ (Figure 3a,b). Detailed physicochemical characterizations are provided in the Supporting Information and Materials and Methods. These materials were exposed to 2D submerged A549 cells pretreated with lipopolysaccharides (LPS) to trigger an initial inflammatory state. We standardized the exposure concentration at 20 μg/mL, a dose has been reported as subcytotoxic across a broad range of mammalian cell lines.^34–36^ To ensure the reliability of our screening, we utilized a size-selected fraction of 100–500 nm. Our data revealed that the elemental composition of MXene is a primary driver of its biological impact. While Ti_3_C_2_ and V_2_C markedly increased IL-8 secretion, potentially exacerbating the inflammatory response, Nb_2_C, Mo_2_C, and Ta_4_C_3_ significantly suppressed IL-8 release (Figure 3c). Among these, Mo_2_C and Ta_4_C_3_ exhibited the most pronounced attenuation, suggesting strong anti-inflammatory potential. To contextualize these specific MXene responses, we included graphene and graphene oxide as benchmark 2D nanomaterials. Given their known anti-inflammatory activity,^37^ they served as a reliable functional reference and both they exhibited potent anti-inflammatory effects within our study. Overall, these comparative data indicate that the specific transition metal used in the MXene lattice plays a decisive role in modulating immunomodulatory effects and biological interactions.

**Figure 3:**
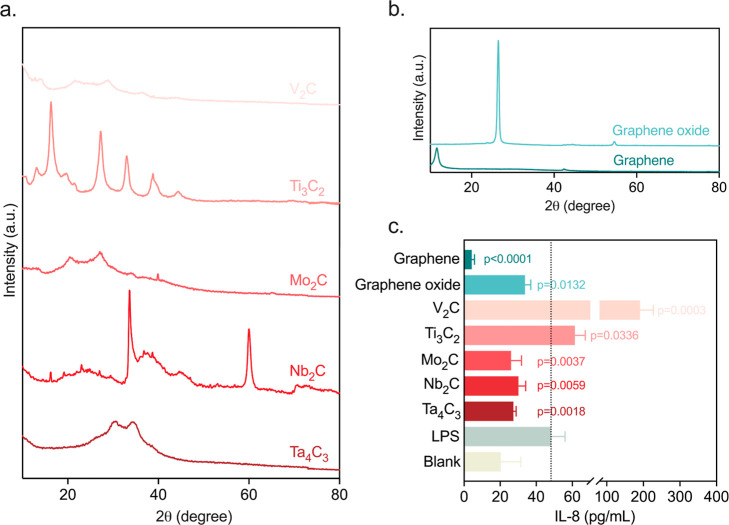
Characterization and systematic evaluation of diverse 2D materials. (a,b) XRD analysis of Ti_3_C_2_, V_2_C, Nb_2_C, Mo_2_C, Ta_4_C_3_, graphene nanosheets, and graphene oxide. (c) Interleukin 8 secretion in 2D submerged A549 cells treated with 20 μg/mL Ti_3_C_2_, V_2_C, Nb_2_C, Mo_2_C, Ta_4_C_3_, graphene nanosheets, and graphene oxide (100–500 nm) following LPS stimulation (10 μg/mL), measured using an ELISA assay. Untreated culture medium served as the blank control while LPS-stimulated cells without additional treatment served as the positive control.

### Cytotoxicity Assessment of Ta_4_C_3_ MXene

While titanium-based MXenes (Ti_3_C_2_T_*x*_) are susceptible to rapid oxidative degradation in physiological environments, tantalum carbide (Ta_4_C_3_) offers a distinct biomedical advantage. Unlike their Ti-based counterparts, Ta-based nanomaterials exhibit superior resistance to oxidative hydrolysis due to the spontaneous formation of a dense, passivating surface oxide layer that acts as a protective shield.^38^ Furthermore, the intrinsic radiopacity of tantalum (*Z* = 73) confers unique theranostic capabilities for simultaneous contrast-enhanced imaging and therapy.^39^ Given the intrinsic material strengths and anti-inflammatory potential observed in our preliminary screen, we shifted our focus toward the safety and biocompatibility of Ta_4_C_3_. As physical dimensions often dictate how a material behaves biologically, we synthesized and characterized size-fractionated Ta_4_C_3_ nanosheets in three ranges: 100–500 nm, 500–2000 nm, and ≥2000 nm. Transmission electron microscopy (TEM) confirmed the characteristic 2D morphology and flake sizes for all three fractions (Figure 4a). A primary challenge with MXenes in aqueous environments is their well-documented susceptibility to oxidative degradation and structural instability.^40^ We first assessed the colloidal behavior of size-fractionated Ta_4_C_3_ by monitoring their zeta potential across various media (Figure 4b). While all particles maintained a net negative surface charge, the magnitude of this charge was modestly attenuated in complex culture media (MEM and RPMI-1640) relative to distilled water and PBS. This indicates that the higher ionic strength and the formation of a protein corona partially screen the inherent surface charge, a physiological adaptation that likely facilitates closer biological interaction between the nanosheets and negatively charged cell membranes. Critically, to validate that these materials remain well-dispersed throughout our experimental exposure windows, we rigorously monitored their stability over a 48 h incubation period in the culture environment. TEM imaging confirmed that all three size fractions preserved their structural integrity and lateral dimensions over time (Figure 4c). This morphological preservation was quantitatively corroborated by a dynamic light scattering (DLS) analysis. As shown in Figure 4d, the hydrodynamic diameter profiles across all groups, including the ≥2000 nm fraction, exhibited persistent, almost overlapping size distribution peaks with no time-dependent shifts over the 48 h timeline, confirming sustained colloidal stability. The steady zeta-potential measurements recorded across all groups at 0, 24, and 48 h (Figure 4e) indicate that the overall surface chemistry of the Ta_4_C_3_ remained stable.

**Figure 4:**
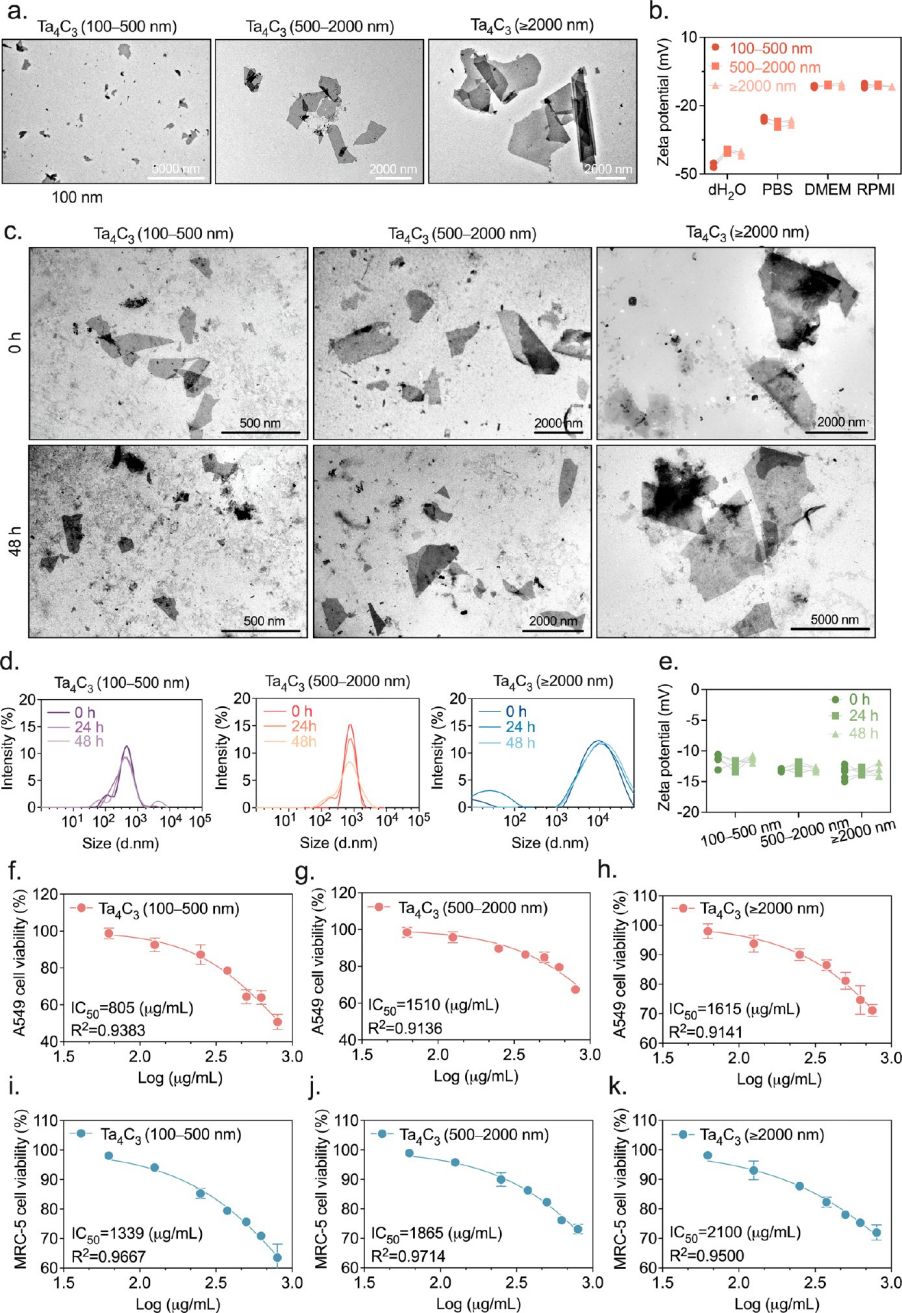
Physicochemical characterization, colloidal stability, and size-dependent cytotoxicity of Ta_4_C_3_ nanosheets. (a) Representative TEM micrographs of delaminated Ta_4_C_3_ nanosheets into three lateral size fractions: 100–500 nm, 500–2000 nm, ≥2000 nm. (b) Zeta potential of the three size fractions in dH_2_O, PBS, DMEM, and RPMI-1640 medium. (c) TEM micrographs of Ta_4_C_3_ nanosheets at 0 h and after 48 h incubation in cell culture medium (DMEM/RPMI 1640 (1:1, v/v)) during the cellular exposure period. (d) DLS size distribution profiles and (e) zeta-potential stability of the three fractions over 48 h in culture medium (DMEM/RPMI 1640 (1:1, v/v)). (f–h) Dose–response viability curves for A549 epithelial cells and (i–k) MRC-5 fibroblasts after 48 h exposure to Ta_4_C_3._ Half maximal inhibitory concentration (IC_50_) values and *R* ^2^ coefficients are indicated. Data are presented as mean ± SD (*n* = 6).

With the colloidal stability established, we evaluated the cytotoxic impact of these size-fractionated Ta_4_C_3_ on A549 and MRC-5 cells using a Calcein-AM assay across a broad concentration range from 62.5 to 800 μg/mL. This range was selected to capture both the potential therapeutic range and extreme overload conditions, allowing us to define clear safety margins. Both cell types showed a concentration-dependent decrease in viability after 48 h of exposure (Figure 4f–k). The smallest nanosheets (100–500 nm) were the most cytotoxic, likely due to their higher surface area-to-volume ratio and increased potential for the cellular uptake. For A549 cells, the IC_50_ for the smallest fraction was 805 μg/mL (95% CI: 779.9–907.6 μg/mL), while the larger fractions yielded significantly higher values, 1510 μg/mL for the 500–2000 nm portion (95% CI: 1307–1811 μg/mL) and 1615 μg/mL for the ≥2000 nm (95% CI: 1379–1967 μg/mL) group. MRC-5 cells followed a similar trend but were notably more resilient, the IC_50_ for the smallest 100–500 nm fraction was 1339 μg/mL (95% CI: 1222–1488 μg/mL), followed by 1865 μg/mL (95% CI: 1676–2107 μg/mL) for the 500–2000 nm portion and 2100 μg/mL (95% CI: 1806–2515 μg/mL) for the ≥2000 nm group.

Even at a massive 800 μg/mL “overload” concentration, cell viability remained above 50% for the most sensitive fraction (100–500 nm) and exceeded the 70% noncytotoxic threshold for the larger fractions (500–2000 nm and ≥2000 nm), as specified by the international standards (ISO 10993-5)^41^ (Supporting Information Figure 3a–f). This proves that Ta_4_C_3_ has a very wide safety window. Based on these results a “green zone” was defined between 0 and 250 μg/mL, where viability consistently exceeds 90%. By anchoring our subsequent functional experiments within this window, we ensured that the observed effects were true therapeutic responses rather than artifacts of cellular distress or “death signals” from dying cells.

### Intracellular Uptake and Fate of Ta_4_C_3_ Nanosheets in A549 Cells

Submicrometer particles (<1 μm) penetrate deep into the lung and reach the alveolar region, where they interact directly with alveolar epithelium.^42^ While the optimal range for inhalation therapeutics typically falls between 1 and 3 μm,^43^ nonphagocytic cells like A549 lung epithelial cells can readily internalize smaller particles.^44^ To map the intracellular fate of Ta_4_C_3_, we utilized TEM to pinpoint the location of the three size fractions within the cellular architecture. For these observations, we selected a concentration of 150 μg/mL. This dose provided enough material for clear visualization under TEM while remaining within the 90% metabolic viability window identified in our initial cytotoxicity test.

However, high-resolution TEM revealed that metabolic stability does not necessarily mean an absence of cellular impact. Compared to cells without Ta_4_C_3_ treatment (Figure 5a), A549 cells exposed to these size-fractionated of Ta_4_C_3_ for 48 h exhibited distinct strip- or circle-shaped structures dispersed throughout the cytoplasm and organelles (Figure 5b–d). Notably, these structures were entirely absent from the nucleus (blue asterisks in the main cell images). To verify that these features were indeed Ta_4_C_3_ nanosheets, we performed energy-dispersive X-ray (EDX) analysis on cells treated with the 100–500 nm and ≥2000 nm fractions (Figure 5e–h). Elemental mapping confirmed the presence of tantalum (Ta) in both intracellular and extracellular compartments (Supporting Information Figure 4b,c), mirroring the composition of the raw particles (Supporting Information Figure 4d). While uptake was evident across the board, the largest flakes (≥2000 nm) showed markedly reduced internalization compared to the smaller fractions. The entry of Ta_4_C_3_ appears to be driven by active membrane dynamics. Near the cell surface, numerous slender cytoplasmic projections—microvilli or filopodia—were observed (red arrows) “trapping” the nanosheets in their vicinity (black arrows) (Supporting Information Figure 4a). These projections facilitate the engulfment of Ta_4_C_3_ into small single-membrane vacuoles. Certain vacuoles in Figure 5j, which generally appeared empty and lacked organelle debris, were identified as lysosomes (red arrows). We also observed larger single-membrane vacuoles (white arrows) resembling autolysosomes, suggesting the activation of the autophagic pathway.^45^ While internal vesicles (black asterisks) might represent autophagic bodies formed after fusion with lysosomes, the typical double-membrane structure of autophagosomes^46^ was not identified, likely obscured by the dense accumulation of Ta_4_C_3_. The internalization of these nanosheets coincided with several ultrastructural signs of toxicity: disrupted organelle integrity, a noticeable loss of lamellar bodies, and hallmarks of apoptosis. Furthermore, extensive vacuolization and autophagic activity led to a visible increase in the overall cell size. These findings, illustrated in the proposed internalization model in Figure 5i, suggest that while Ta_4_C_3_ is largely biocompatible at lower doses, high-concentration uptake triggers a cascade of cellular stress and structural damage.

**Figure 5:**
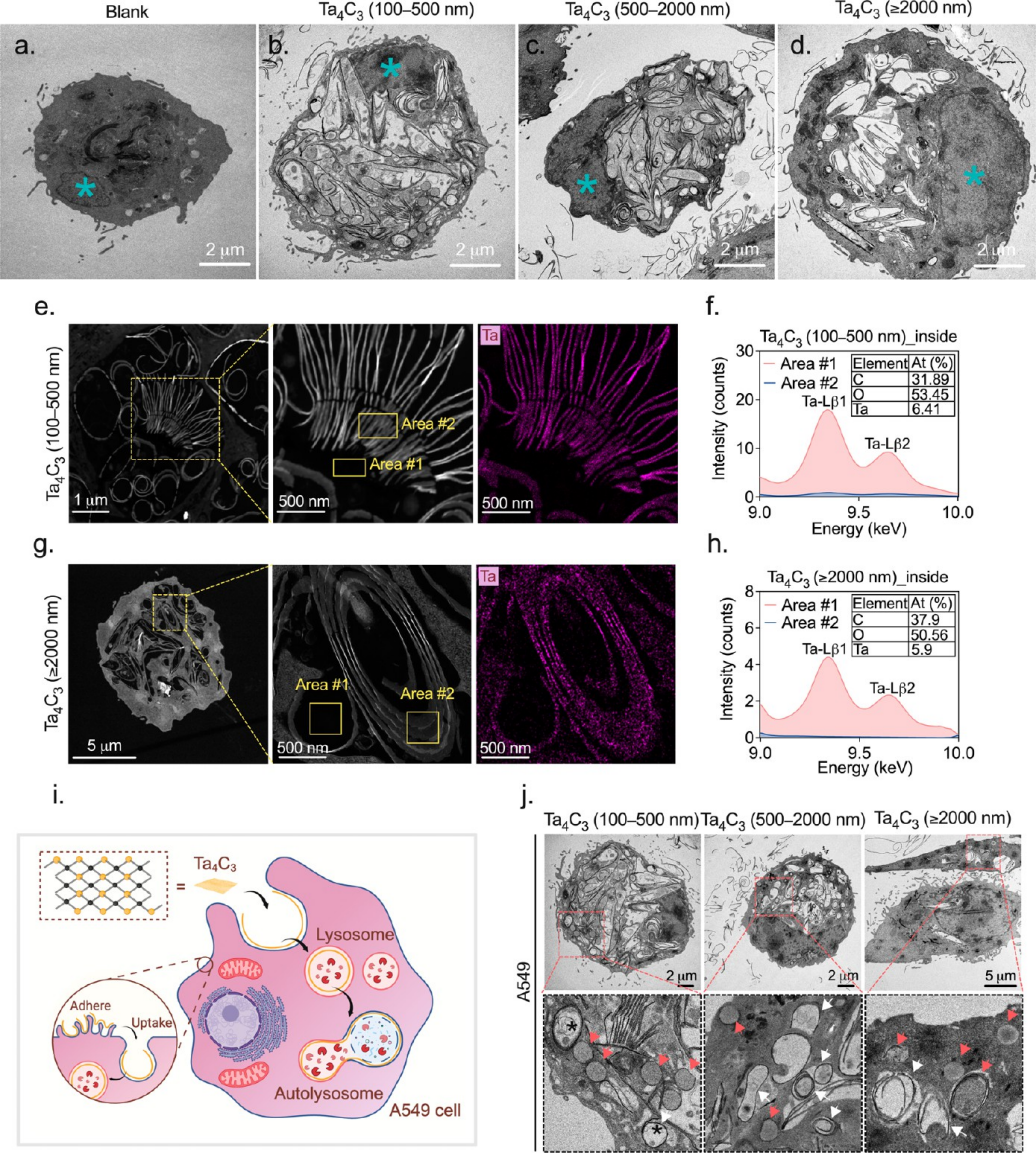
Intracellular trafficking and chemical identification of internalized Ta_4_C_3_ MXene in A549 epithelial cells. (a–d) Representative bio-TEM micrographs (acquired using a Talos L120C microscope) of A549 cells following 48 h exposure to 150 μg/mL size-fractionated Ta_4_C_3_ nanosheets. Cells without Ta_4_C_3_ treatment (a) serve as the blank control; blue asterisks indicate nuclei. Internalized nanosheets appear as high-electron-density strip- and circle-shaped structures distributed throughout the cytoplasm. (e–h) EDX spectroscopy and high-angle annular dark-field (HAADF-STEM) elemental mapping of intracellular Ta_4_C_3_ (100–500 nm and ≥2000 nm fractions) were performed using a Talos F200X S/TEM. Magenta mapping and localized spectral analysis confirm the presence of tantalum (Ta-Lβ1and Ta-Lβ2 peaks) within the identified cytoplasmic structures. Spectral intensity was derived by background subtraction (area #1 vs area #2). (i) Schematic illustration of the proposed endocytic internalization and lysosomal trafficking pathway for Ta_4_C_3_ in A549 cells. (j) High-magnification TEM micrographs identifying Ta_4_C_3_ sequestration within single-membrane vesicles, including lysosomes (red arrows) and autolysosomes (white arrows). Black asterisks indicate autophagic bodies containing degraded cytoplasmic cargo.

### Anti-inflammatory Effects Observed upon Treatment with Ta_4_C_3_ MXene

To move beyond the safety profiles of Ta_4_C_3_ and into the territory of functional performance, we challenged the material within an organotypic 3D ALI environment. This system was selected because the alveolar epithelium acts as the absolute frontline against inhaled threats and any meaningful therapeutic must prove its worth where the battle actually begins. To replicate the violent, epithelial-driven inflammatory stimuli seen in conditions like acute respiratory distress syndrome,^47^ we established an acute lung injury model utilizing LPS (10 μg/mL) as a gold-standard inflammatory trigger. This pathological stress test serves as a rigorous testing ground to determine whether Ta_4_C_3_ can truly restore homeostasis to a compromised lung barrier.

To rule out experimental artifacts, all materials were verified as endotoxin-free via LAL testing prior to cell exposure (Supporting Information Figure 2). We initiated a pilot dose-dependent trial to compare the anti-inflammatory efficacy across culture models of increasing biological complexity. As shown in Supporting Information Figure 5a–e, traditional 2D submerged cultures exhibited a low basal cytokine signal, limiting their ability to capture dynamic inflammatory responses. In contrast, 3D ALI coculture models showed a more responsive cytokine release profile, supporting their suitability for immunomodulatory assessment. With the 3D ALI model established, we next evaluated the dose-dependent efficacy of Ta_4_C_3._ While both 20 and 50 μg/mL reduced IL-6 and IL-8 levels, the 50 μg/mL concentration produced a more pronounced anti-inflammatory effect. As this higher concentration had previously been shown to fall within a biocompatible range during our earlier safety evaluations, the 50 μg/mL dose was selected for all subsequent investigations.

Even a 3D coculture remains a shadow of the in vivo environment if it lacks the immune system’s frontline defenders. For this reason, we advanced to a 3D ALI triculture model, introducing THP-1-derived macrophages into the A549/MRC-5 bilayer to capture the missing link: the epithelial-immune crosstalk. The experimental timeline in Figure 6a depicts the days of cell seeding, treatments, and sample collection, constituting a 19 day protocol that allows for full barrier differentiation and macrophage attachment before Ta_4_C_3_ treatment. The basolateral medium samples collected after 48 h treatment with Ta_4_C_3_ on day 19 was chosen for analysis.

**Figure 6:**
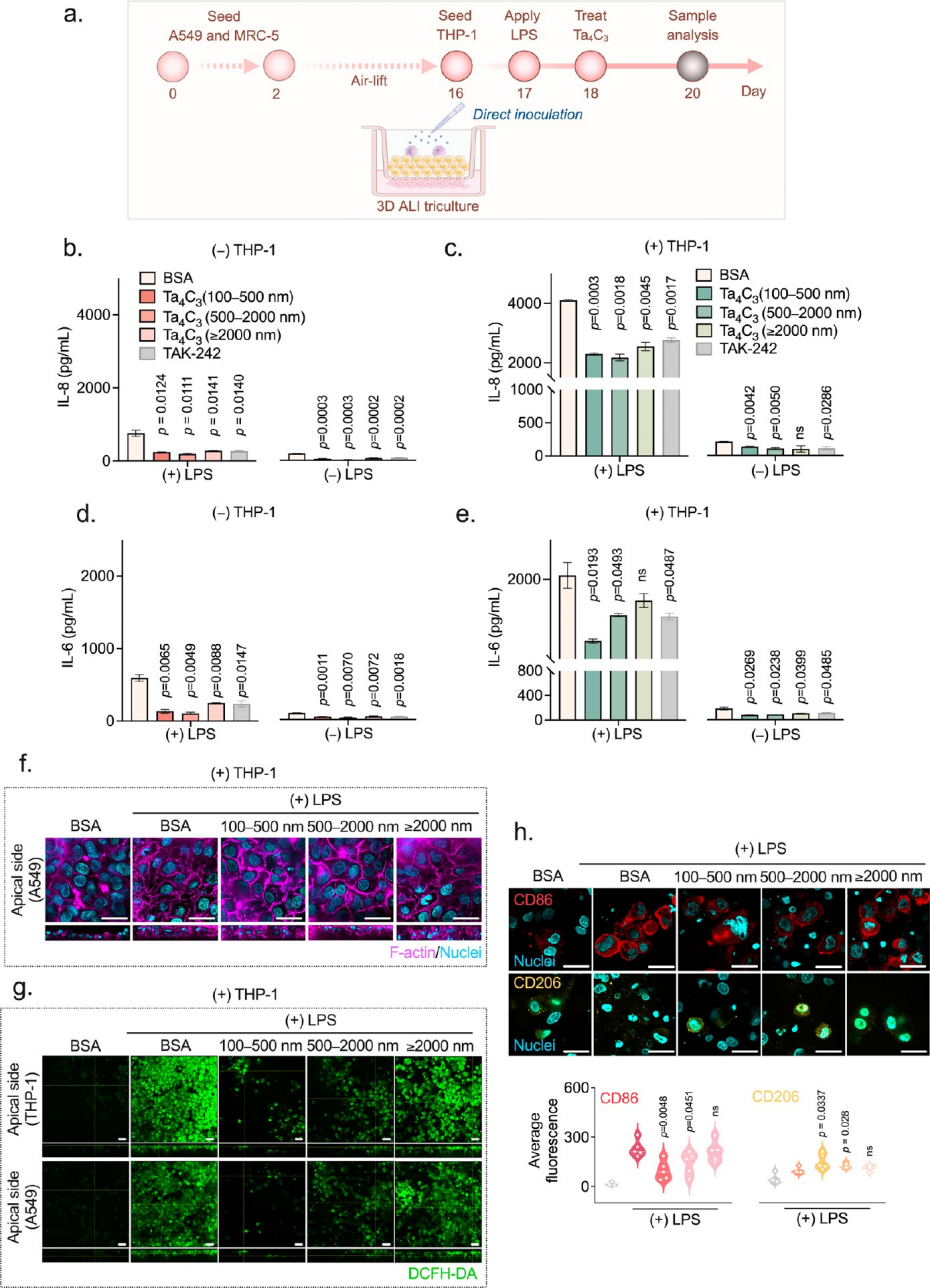
Size-dependent anti-inflammatory efficacy of Ta_4_C_3_ in a 3D lung acute injury model. (a) Experimental timeline for the establishment of the LPS-induced acute lung injury model. Sequential development of the 3D lung triculture includes air-lift to initiate the ALI for 14 days (day 1–15), PMA-differentiated THP-1 seeding (day 15), LPS stimulation (1 μg/mL, day 16), and therapeutic intervention with size-fractionated Ta_4_C_3_ nanosheets (50 μg/mL) (day 17). (b–e) Pro-inflammatory cytokine secretion profiles for IL-8 (b,c) and IL-6 (d,e) in the absence (−) and presence (+) of differentiated THP-1 macrophages. Tissues treated only with BSA served as the negative control, LPS-stimulated tissues without Ta_4_C_3_ treatment served as the positive control, and TAK-242-treated tissues served as the positive therapeutic control. (f) Orthogonal SDCM images showing F-actin (magenta) and nuclei (cyan) morphology of A549 epithelial layers in the (+) THP-1 model following LPS and Ta_4_C_3_ treatment. (g) Orthogonal SDCM views of DCFH-DA staining (green) within apical macrophages and epithelial layers. (h) Representative immunofluorescence images and corresponding quantification of M1 (CD86, red) and M2 (CD206, yellow) markers in THP-1 macrophages. *P* values were calculated by the unpaired *t*-test (ns = not significant). Data represent mean ± SD (*n* = 3 independent biological replicates). Scale bars: 20 μm.

IL-6 and IL-8 are proinflammatory mediators that play important roles in local injury and inflammatory reactions in the development of human pulmonary diseases. The release of these cytokines was assessed by enzyme-linked immunosorbent assay (ELISA) on 3D ALI cultures with (+) and without (−) THP-1 cells, which were prestimulated with LPS (positive control) and subsequently treated with either Ta_4_C_3_ nanosheets or the TAK-242 (positive therapeutic control).

In (−) THP-1 cocultures, LPS stimulation induced a statistically significant but moderate increase in IL-8 and IL-6 release compared to the tissues treated only with BSA (negative control). Following exposure to Ta_4_C_3_, these cocultures exhibited a statistically significant decrease in all investigated cytokines. The intermediate 500–2000 nm sheets induced a suppression more pronounced than that of the other two fractions (Figure 6b,d). This suppressive effect extended beyond inflamed conditions. In cocultures without LPS stimulation, Ta_4_C_3_ alone also reduced baseline IL-6 and IL-8 levels, demonstrating an inherent capacity to dampen baseline inflammation. On the other hand, the immune-competent (+) THP-1 tricultures exhibited a highly amplified reaction to LPS, with both IL-8 and IL-6 secretion surging nearly 4-fold compared to the THP-1-deficient cocultures. Under these conditions, the size-dependent response to Ta_4_C_3_ shifted, and the 100–500 nm fraction produced the most pronounced reduction in cytokine release, exceeding the effect observed for the selective TLR4 inhibitor^48^ TAK-242 (Figure 6c,e). These results indicate that the presence of macrophages alters both the magnitude of the inflammatory response and the relative efficacy of Ta_4_C_3_ size fractions.

(−) THP-1 cocultures and (+) THP-1 tricultures were treated with size-fractionated Ta_4_C_3_ (50 μg/mL) following LPS stimulation and imaged using SDCM to observe cell morphology. In both models, LPS (10 μg/mL) treatment led to a slight change in A549 cells: they lost their regular shape and became disorganized, which caused discontinuity of the cellular layer. On the other hand, partially reversed morphological changes were observed in the Ta_4_C_3_-treated tissues, especially for the 100–500 nm fraction in (+) THP-1 tricultures (Figure 6f) and the 500–2000 nm fraction in (−) THP-1 cocultures (Supporting Information Figure 6).

The production of reactive oxygen species (ROS) is a critical biomarker for evaluating the biological impact of nanomaterials on the respiratory barrier. Previous research suggests that MXenes can alleviate ROS-induced inflammation in conditions like intestinal and pancreatic inflammation due to their antioxidant properties.^49,50^ To assess the response of (+) THP-1 triculture model exposed to LPS, we monitored the oxidation of the DCFH-DA dye as a direct indicator of ROS accumulation. As shown in Figure 6g, LPS stimulation triggered a widespread oxidative burst, characterized by a sharp surge in green fluorescence across both the apical THP-1 macrophages and the underlying A549 epithelial layers. A culture model comparison study reported that differentiated THP-1 cells grown under submerged conditions exhibited increased ROS production across all tested models, including monocultures, cocultures and tetra-cultures, in response to an oxidative stimulus 2,2′-azobis-2-methyl-propanimidamide-dihydrochloride (AAPH).^51^ Ta_4_C_3_ intervention markedly reduced the size-dependent LPS-induced ROS in a size-dependent manner. While all fractions reduced the signal compared with the tissues treated only with BSA (negative control), the 100–500 nm fraction proved superior. Conversely, in the (−) THP-1 coculture model (Supporting Information Figure 7), the 500–2000 nm fraction emerged as the most effective regulator of oxidative stress. These results indicated that Ta_4_C_3_ (<2 μm) nanosheets were able to effectively scavenge ROS and protect the alveolar interface from the oxidative damage that drives pro-inflammatory cytokine production. To determine if the reduction in oxidative stress translated into a functional shift in immune behavior, we characterized the macrophage phenotype using SDCM. The triculture models were dual-stained for CD86 (red), a marker for pro-inflammatory M1 activation, and CD206 (yellow), a marker for pro-healing M2 polarization (Figure 6h). In the LPS-treated group, the apical surface was dominated by an intense CD86 signal, with macrophages appearing densely clustered and highly activated, while the CD206 signal remained markedly weak, indicating M1 macrophage activation. Treatment with Ta_4_C_3_ fractions induced a visible “color switch” in the immune landscape. This phenotypic shift was most evident with the 100–500 nm fraction, where the CD86 red fluorescence was dramatically attenuated and replaced with a robust CD206 signal. These visual data provided direct evidence that Ta_4_C_3_ did not merely suppress inflammation but actively facilitated a transition from a pro-inflammatory M1 to a pro-healing M2 phenotype.

### Antifibrotic Effects Observed Treatment with Ta_4_C_3_ MXene

Building upon the demonstrated anti-inflammatory and immunomodulatory efficacy of Ta_4_C_3_, we further investigated its potential to combat the fibrotic cascade. Given the close pathological correlation between chronic inflammation and fibrotic disease progression,^52^ where persistent M1-driven oxidative stress often triggers tissue scarring, we examined whether the suppression of these inflammatory mediators could similarly interrupt the fibrotic process. Pulmonary fibrosis is characterized by the excessive deposition of extracellular matrix (ECM) components, primarily driven by FMT and EMT.^53,54^ As detailed in Figure 7a, we established a comprehensive 19 day 3D ALI triculture protocol, introducing TGF-β (50 ng/mL) on day 16 as a potent inducer of ECM production to simulate the fibrotic environment.^55^ To establish a baseline for therapeutic efficacy, we first evaluated the concentration (50 μg/mL) previously utilized for anti-inflammatory assay within both 3D ALI coculture (Supporting Information Figure 8a,b) and 2D submerged culture systems (Supporting Information Figure 8c,d). The initial trials showed that 50 μg/mL was insufficient to inhibit the robust fibrotic response induced by TGF-β even though it was effective in reducing pro-inflammatory cytokine release. These findings indicate that the dose needed to reverse fibrotic tissue remodeling was far higher than that required to treat acute lung inflammation, leading us to investigate increased concentrations to achieve a functional antifibrotic effect. To identify a more effective therapeutic window, we explored the dose-dependent effect at 150 μg/mL and 200 μg/mL within both 2D and 3D ALI coculture systems, as shown in Supporting Information Figure 9. While the 150 μg/mL dose began to show discernible inhibitory effects, it was the 200 μg/mL concentration that consistently achieved statistically significant reductions in fibronectin and pro-collagen I-α1 across both models. Interestingly, the 3D ALI coculture system exhibited a far more robust fibrotic response to TGF-β compared to the 2D submerged monocultures, with significantly higher baseline levels of ECM proteins. The increased sensitivity of ECM protein production in 3D ALI systems makes them a more effective platform for studying pulmonary diseases and therapeutic interventions compared to 2D monocultures. Based on the above-mentioned results, we selected the 200 μg/mL dose to further investigate the antifibrotic capacity of Ta_4_C_3_ within our most complex 3D ALI triculture environments.

**Figure 7:**
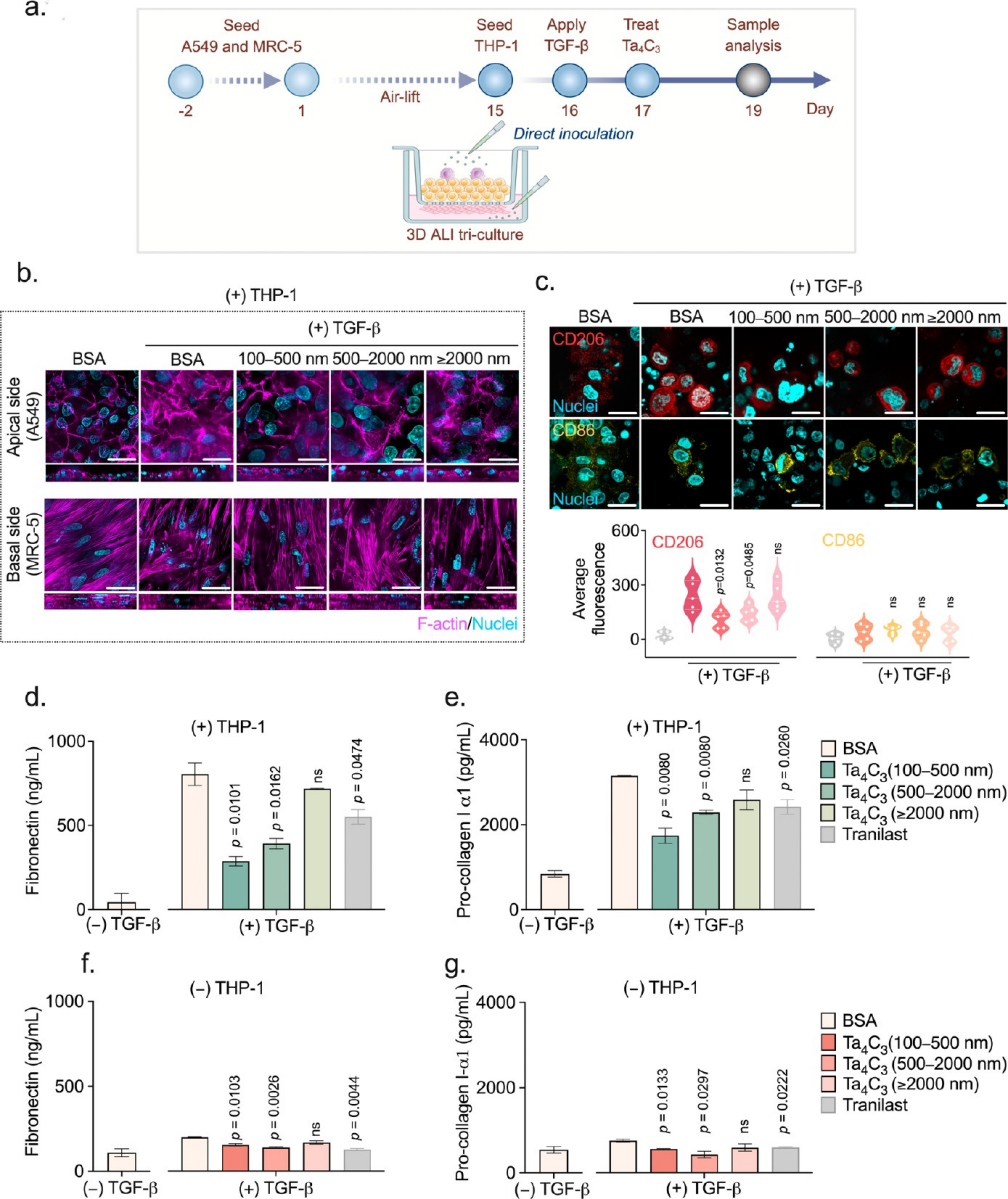
Size-dependent antifibrotic efficacy of Ta_4_C_3_ in a 3D lung fibrosis model. (a) Experimental timeline for the establishment of the TGF-β-induced pulmonary fibrosis model. Following a 14 day air-lift differentiation, the 3D triculture was seeded with PMA-differentiated THP-1 macrophages (day 15), stimulated with TGF-β (50 ng/mL,day 16), and treated with size-fractionated Ta_4_C_3_ nanosheets (200 μg/mL) (day 17). (b) Orthogonal SDCM micrographs of the apical (A549) and basal (MRC-5) layers in the (+) THP-1 model showing F-actin (magenta) and nuclei (cyan). (c) Immunofluorescence micrographs and corresponding quantification of M2 (CD206, red) and M1 (CD86, yellow) markers in THP-1 macrophages. (d–g) Secretion levels of pro-fibrotic markers fibronectin (d,f) and pro-collagen I-α1 (e,g) in models with (+) and without (−) THP-1 macrophages. Tissues treated only with BSA served as the negative control, TGF-β-stimulated tissues without Ta_4_C_3_ treatment served as the positive control, and Tranilast-treated tissues served as the positive therapeutic control. *P* values were calculated by the unpaired *t*-test (ns = not significant). Data represent mean ± SD (*n* = 3 independent biological replicates). Scale bars: 20 μm.

(−) THP-1 cocultures and (+) THP-1 tricultures were treated with size-fractionated Ta_4_C_3_ (200 μg/mL) following TGF-β exposure and imaged using SDCM to observe cell morphology. Overt changes in apical A549 layers and basolateral MRC-5 layers were evident with TGF-β when compared with the tissues treated only with BSA (negative control). A complete EMT was observed in the apical A549 layer: they lost their “cobblestone” shape and formed dense, parallel F-actin stress fibers; FMT was found in the MRC-5 layers: the cytoskeleton was disrupted and lost cell–cell contact. The tissues were observed with contraction and nodule formation, leading to the detachment and loss of cells from the membrane. On the other hand, treatment with Ta_4_C_3_ showed a restored pattern, particularly pronounced for the 100–500 nm fraction in (+) THP-1 tricultures (Figure 7b) and for the 500–2000 nm fraction in (−) THP-1 cocultures (Supporting Information Figure 10). Macrophage phenotype characterization (Figure 7c) showed that TGF-β triggered a shift toward an M2-like state (high CD206, low CD86), due to the fact that TGF-β is a pro-fibrotic driver and acts as a potent suppressor of M1 activation.^56^ Exposure to the 100–500 nm Ta_4_C_3_ fraction led to a marked decrease in CD206 expression while keeping CD86 at baseline levels, suggesting a reversal of the pro-fibrotic macrophage polarization.

Since chronic inflammation can trigger a pro-fibrotic response,^57^ we used ELISA to investigate secretion of the pro-fibrotic markers on 3D ALI cultures which were prestimulated with TGF-β (positive control) and subsequently treated with either Ta_4_C_3_ nanosheets or the Tranilast (positive therapeutic control). A significant increase of fibronectin and pro-collagen I-α1 was observed after treatment with TGF-β in (+) THP-1 tricultures, compared to the tissues treated only with BSA (negative control) (Figure 7d,e). Conversely, size-fractionated Ta_4_C_3_ resulted in a size-dependent reduction in these pro-fibrotic markers. The smallest MXene fraction (100–500 nm) demonstrated a potency comparable to that of Tranilast. Tranilast is an antiallergy drug, which suppresses TGF-β expression and inhibits fibrosis effects.^58^ This suggests that as-synthesized MXenes, when tailored to specific nanoscale ranges, could serve as versatile platforms for targeted pulmonary therapies. At the same time, although antifibrotic effects were also observed in the (−) THP-1 cocultures particularly from 500 to 2000 nm fraction (Figure 7f,g), the presence of macrophage in the (+) THP-1 tricultures elicited a markedly stronger fibrotic response when treated with TGF-β.

Previous research has highlighted the existence of epithelial-fibroblast crosstalk within ALI multilayered tricultures.^28^ In our study, the 10 μm thick PET membrane of the Transwell inserts creates a substantial physical barrier between the A549 epithelial layers and MRC-5 fibroblast layers. However, direct cellular contact is maintained through the membrane pores, as visualized by SDCM, showing that several F-actin filaments extended through the pores of the PET membrane, bridging the apical and basolateral compartments (Supporting Information Figure 11). These visual data prove that the model is not merely a collection of three cell types in a single well but a functionally integrated tissue where cells physically communicate. This partial cell-to-cell contact is vital for fibrotic modeling as the direct interaction between these cell types is known to drive fibroblast activation and ECM remodeling in vivo. The observed physical integration supports the hypothesis that the macrophage-mediated inflammatory-to-fibrotic transition is driven by a direct cellular interplay.^59^ Such multicellular interactions and physical cell–cell contacts are critical for the development of structural alterations that occur within the epithelial-EMTU following the inhalation of fibrous nanomaterials.^60^

## Discussion

A primary consideration in our experimental design was the focus on the MXene size fraction in the 100–500 nm range when prescreening diverse MXenes. In addition to toxicological concerns observed in the sub-100 nm fraction,^61^ isolating a high purity population of these ultrafine MXene nanosheets remains technically challenging. In practice, achieving these ultrafine dimensions typically requires prolonged high-power probe sonication or aggressive mechanical shearing, processes that substantially reduce the yield of pristine material and markedly increase both energetic and material costs.^62^ The intensive processing required for sub-100 nm scales compromises the MXene lattice, creating a high density of defects and a larger edge-to-basal-plane ratio.^63^ Because degradation typically begins at these high-energy sites, these ultrafine flakes become particularly susceptible to rapid oxidation under physiological conditions.^64^ As degradation proceeds, the shifting material composition makes it increasingly difficult to isolate the biological effects of pristine MXene from its oxidation products. Consequently, focusing on the 100–500 nm range provides a structurally stable and reliable baseline, allowing us to evaluate the intrinsic immunomodulatory potential of the pristine material.

The shift in the optimal therapeutic size fraction observed with increasing cellular model complexity represents a key outcome of this study. In the epithelial-fibroblast cocultures, the 500–2000 nm fraction initially appeared effective; however, the addition of macrophages in our triculture system revealed the 100–500 nm fraction as a more potent regulator of the inflammatory microenvironment. This model upgrade amplifies the sensitivity of the model to smaller nanosheets, which more efficiently promote the transition from pro-inflammatory M1 to pro-healing M2 macrophages. This observation is consistent with foundational studies demonstrating that inclusion of immune cells in 3D lung models enables detection of particle-induced cellular responses that are not captured in monocultures.^65^ Without the immune component, these subtle but critical structure–activity relationships may remain obscured, potentially leading to the selection of less effective therapeutic candidates during preclinical development. This enhanced sensitivity of the model likely arises, at least in part, from the differential uptake capacities of the cell types involved. In epithelial cells, uptake is largely restricted to particles below ∼300–500 nm and occurs predominantly via clathrin-mediated endocytosis, whereas macrophages efficiently internalize nanosheets across a broader size range through macropinocytosis and phagocytosis, thereby enhancing sensitivity to size-dependent effects.^44,66^ This distinction explains the diminished therapeutic potency of larger nanosheets (>2000 nm), whose lateral dimensions may exceed the phagocytic capacity of macrophages, resulting in frustrated phagocytosis. Conversely, smaller nanosheets (100–500 nm) remain accessible to multiple endocytic uptake mechanisms.

Once internalized, the high surface-area-to-volume ratio of the 100–500 nm Ta_4_C_3_ fraction allows for scavenging cellular ROS and damping of a robust M1 polarization. The triculture model enables epithelial–immune–mesenchymal crosstalk where macrophages act as signal integrators, amplifying subtle epithelial cues into a broad anti-inflammatory response. Our data establish a universal 2000 nm lateral size as a critical threshold for Ta_4_C_3_ pulmonary efficacy. Nanosheets exceeding this dimension are internalized less efficiently, which corresponds to a decrease in the therapeutic activity. As visualized by our cryo-TEM and EDX analyses within A549 cells, the biological activity appears dependent on lysosomal sequestration and potentially autophagy.^67^ The 100–500 nm Ta_4_C_3_ fraction is efficiently sequestered into autolysosomes and promoted a “cellular cleaning” process.^68^ This direct evidence of the Ta_4_C_3_ uptake—particularly for particles smaller than 2000 nm—and its link to cellular impact provide crucial mechanistic insight. This finding contrasts with various reports for other MXene compositions; for instance, while Ti_3_C was shown to be readily internalized by immune cells, V_4_C_3_ was not, possibly due to vanadium counteracting phagocytic activity.^12^ Our results with Ta_4_C_3_ thus reinforce the emerging understanding that altering the chemical composition of MXenes can profoundly impact the cellular uptake efficiency by different cell types and, consequently, their subsequent biological effects. While the triculture remained functionally intact throughout our experiments, a dedicated assessment of high-resolution internalization kinetics and cell-specific cytotoxicity within the THP-1 macrophage population remains a priority for future investigation. This will further refine the mechanistic understanding of how immune cells process these 2D materials in comparison to their structural counterparts.

Beyond biological efficacy, a major challenge in current MXene research is the lack of harmonized guidance on sample characterization and dosimetry, which often leads to inconsistent toxicity data across studies. As recently noted by the OECD’s Early4AdMa assessment,^69,70^ many reports provide insufficient detail regarding material dispersion, casting doubt on the adequacy of their toxicological findings. The thin platelet morphology of 2D Ta_4_C_3_ presents additional challenges for stable dispersion as its colloidal behavior is often more complex than that of traditional spherical inorganic nanomaterials. Standard techniques such as DLS rely on spherical geometry assumptions, a limitation clearly noted in international standards (ISO, 2023).^71^ Accordingly, DLS data must be interpreted alongside direct imaging methods, such as TEM, when assessing the physical state of micron-scale MXene flakes.^72^ In this study, we used DLS primarily to track relative stability of the nanosheets in biological media rather than as an absolute measure of particle size. Using this multiparametric approach, we confirmed that the smallest fraction (100–500 nm) maintained a stable hydrodynamic diameter profile under exposure conditions. This verification supports the idea that the cells in our assays were exposed to a consistent and well-defined dose of dispersed nanosheets.

Following this physical standardization, we defined a biological therapeutic window to avoid concentration-dependent cytotoxicity that could confound the immunomodulatory readouts. Accordingly, the delivered dose was carefully calibrated relative to basal cellular tolerance. In the acute lung injury model, the anti-inflammatory dose (50 μg/mL) maintained cell viability near 100% (Supporting Information Figure 4), providing a 16-fold safety margin relative to the IC_50_. In the pulmonary fibrosis model, a higher antifibrotic dose (200 μg/mL) was used, which remained within the high-viability range (>90%) for the primary structural barrier. By following rigorous reporting standards, we ensured that the observed biological responses reflect genuine therapeutic effects rather than artifacts of material-induced stress.

## Study Limitations

This study was designed to mimic the pulmonary microenvironment and predict the therapeutic potential of MXene materials in a well-established 3D ALI system. However, 3D ALI systems offer significantly advanced performance over traditional 2D submerged systems. Several factors must be considered before these findings can be translated to clinical application. (i) Lack of long-term fate studies. We used immortalized cell lines to assemble our model because they offer a stable, cost-effective, and easy-to-handle system with the experimental flexibility needed for rapid prescreening. However, these cell lines are not ideal for assessing persistent inflammation and the cell matrix interactions that predict chronic pulmonary fibrosis. While commercially available primary models like the EpiAlveolar model allow for repeated exposures over 3 weeks, our cell-line model is only stable for 3–4 days.^24,73^ A549 cell line fails to form sophisticated tight junctions required to create an intact epithelial barrier. Unlike primary alveolar type II cells, which develop high TEER overtime,^74^ the relatively “leaky” nature of our model barrier may compromise the assessment of long-term fate and translocation of MXenes. (ii) Cell line metabolic activity. A549 cells have very high endocytic activity likely because they are derived from adenocarcinomas.^66^ This might result in the MXene uptake in our model being higher than what would be observed in quiescent primary epithelial cells in vivo. Our model might be “hungrier” for particles than a healthy human lung, which could influence the observed therapeutic threshold. (iii) Absence of the endothelium. A major architectural gap in this model is the lack of a microvascular endothelial layer. In the real lung, the “blood-air barrier” is a three-way conversation between the epithelium, the interstitium, and the endothelium.^75^ The missing capillary side means we are not accounting for how blood flow, nutrient exchange, or endothelial signaling influences the fibrotic process. (iv) The static environment. Our system is static. In a living lung, the tissue is constantly pulled and stretched by breathing. This mechanical tension is a massive driver for turning fibroblasts into myofibroblasts. Without that cyclic stretch, we might be underestimating the total fibrotic stimulus against which the MXenes are actually working against. (v) Anatomical relevance: in the native lung, the alveolar region is defined by an ultrathin, monolayered barrier optimized for gas exchange. Our model exhibits a multilayered epithelial structure that more closely resembles the complexity of the bronchial-interstitial environment or a thickened, pathological alveolar state rather than a healthy alveolar membrane. This stratified organization is a known characteristic of A549-based cell cultures at the ALI.^76^ While this departure from native alveolar thinness is a limitation, the resulting structure provides a rigorous biological barrier to evaluate the penetration and localized efficacy of inhalable nanotherapeutics. (vi) Biological relevance. No in vitro system can replicate systemic factors like lymphatic drainage or multiorgan crosstalk. While our 3D model offers screening function, in vivo studies are ultimately required to confirm the safety and efficacy of these MXenes in a full physiological context, especially in consideration of the complex clearance and biopersistence of high-aspect-ratio nanomaterials.

## Conclusions

In summary, this study establishes Ta_4_C_3_ MXene as a multifunctional nanotherapeutic, with efficacy strongly dependent on a 2000 nm lateral size threshold. While simpler cocultures suggested a broader therapeutic range, the immunocompetent triculture demonstrated that the 100–500 nm fraction offered superior protection by effectively engaging immune cells. By bridging the gap between material synthesis, detailed physicochemical characterization, and advanced microphysiological evaluation, this work provides a foundational preclinical basis for Ta_4_C_3_ as a next-generation inhalable agent for complex lung diseases.

## Materials and Methods

### Synthesis of Ta_4_C_3_ MXene

Delaminated, few-layered Ta_4_C_3_ MXene nanosheets were fabricated via selective etching and a liquid-phase exfoliation process. Briefly, 2 g of Ta_4_AlC_3_ MAX phase powder was slowly added to 40 mL of 49% hydrofluoric acid (HF) within a poly­(tetrafluoroethylene) reaction vessel. The etching process was maintained at 45 °C for 96 h under continuous magnetic stirring at 500 rpm. The resulting suspension was washed repeatedly with deionized (DI) water and ethanol through centrifugation (7000 rpm, 5 min per cycle) until the supernatant reached a pH ≥ 6. To achieve delamination, the multilayered Ta_4_C_3_ precipitate was redispersed in 100 mL of deoxygenated DI water and subjected to probe sonication at 500 W for 1 h. During sonication, the vessel was chilled in an ice bath and purged with a continuous N_2_ flow to prevent oxidative degradation. The final suspension was centrifuged at 3000 rpm for 30 min to remove unetched MAX phase and multilayered residues. The supernatant, containing delaminated Ta_4_C_3_ MXene flakes, was collected and stored under an inert atmosphere at 4 °C for subsequent use. To obtain Ta_4_C_3_ nanosheets with defined lateral dimensions, the as-synthesized polydisperse suspension was processed via liquid cascade centrifugation. This successive sedimentation approach allowed for the isolation of three distinct size regimes: 100–500 nm, 500–2000 nm, and ≥2000 nm. Centrifugation was performed by using a fixed-angle rotor at 4 °C to prevent thermal oxidation. The first fraction (≥2000 nm) was collected by centrifuging the initial delaminated supernatant at 1000 rpm for 30 min and recovering the precipitate. The subsequent supernatant was then processed at 3000 rpm for 30 min to isolate the 500–2000 nm fraction. Finally, the remaining supernatant was centrifuged at 8000 rpm for 60 min to yield 100–500 nm nanosheets. Each fraction was redispersed in deoxygenated DI water, and the concentrations were standardized to 5 mg/mL using UV–vis spectrophotometry based on the established extinction coefficient for Ta_4_C_3._

### Characterization of Ta_4_C_3_ MXene Morphology and Colloidal Stability

TEM, DLS, and zeta-potential measurements were employed to characterize the size-fractionated Ta_4_C_3_ samples under both stock and experimental conditions. TEM was used to characterize the morphology and lateral dimensions of the three size-fractionated Ta_4_C_3._ For stock solution characterization, the samples were redispersed in DI water to a concentration of 500 μg/mL to establish baseline properties. For experimental characterization, the samples were diluted in DMEM/RPMI 1640 (1:1, v/v) medium at a working concentration of 200 μg/mL and incubated at 37 °C and 5% CO_2_. Colloidal stability and surface charge were evaluated via DLS and zeta potential at 0, 24, and 48 h to monitor time-dependent aggregation and biostability. In parallel, structural morphology was examined by TEM at 0 and 48 h to observe potential flake degradation or protein corona formation. For TEM imaging, to facilitate the electrostatic adsorption of the negatively charged Ta_4_C_3_ nanosheets, carbon-coated copper grids were pretreated with 0.1% poly-d-lysine solution for 5 min to impart a positive surface charge. A 5 μL aliquot of the MXene suspension was deposited onto the grids, incubated for 1 min, and dried before imaging with bright-field TEM (FEI Morgagni 268, USA) operating at 100 kV. Hydrodynamic size and zeta potential were measured using a Malvern Zetasizer Nano ZS equipped with a folded capillary cell. All data are presented as the mean ± SD (*n* = 5 independent biological replicates). X-ray diffraction (XRD) measurement was obtained by the D8 ADVANCE X-diffractometer (Bruker AXS) with Cu Kα radiation (λ = 1.5418 Å) in the range 10° < 2θ < 80° at 40 kV and 35 mA. To minimize oxidation and maintain material integrity for research, Ta_4_C_3_ were stored at 4 °C and used within three months to ensure optimal freshness and stability. Five distinct batches of Ta_4_C_3_ were used throughout this study to ensure reproducibility.

### Cell Cultures

Human lung adenocarcinoma cells (A549 cell line; ATCC CCL-185), human lung fibroblasts (MRC-5 cell line; ATCC CCL-171) and human monocytes (THP-1 cell line; ATCC TIB-202) were obtained from the American Tissue Culture Collection (ATCC) and maintained according to supplier’s instructions. A549 cells were maintained in Dulbecco’s modified Eagle’s medium (DMEM, Gibco, CH) supplemented with 10% (v/v) fetal bovine serum (FBS, Gibco, CH). MRC-5 were cultured in modified Eagle’s medium (MEM, Gibco, CH) supplemented with 10% (v/v) FBS. THP-1 was grown in RPMI-1640 medium with 10% (v/v) FBS. All cell lines were maintained under 5% CO_2_ at 37 °C and 95% humidity.

### Differentiation and Characterization of THP-1 Macrophages

To induce differentiation into macrophage-like cells, THP-1 cells were resuspended at a density of 4 × 10^5^ cells/mL in RPMI-1640 medium supplemented with 20 ng/mL phorbol-12-myristate-13-acetate (PMA) and incubated for 24 h. Following the differentiation period, the PMA-containing medium was replaced with fresh, PMA-free complete medium for an additional 24 h to allow the cells to reach a stable macrophage-like state before addition in the tricultures. PMA was prepared as a 10 mg/mL stock solution in anhydrous dimethyl sulfoxide (DMSO), stored at −20 °C in the dark, and diluted immediately before use. To harvest the cells, the differentiated macrophages were rinsed with PBS and detached using accutase. To ensure complete removal of residual PMA, the detached cells were centrifuged and washed twice with PBS.

### 3D ALI Multilayered Tricultures

To produce the tricultures, on day 2, MRC-5 cells were seeded onto the basolateral side of inserts coated with poly-d-lysine. Briefly, microporous polyethylene (PET) membrane inserts (0.4 μm pore size, pore density 2 × 10^6^ pores/cm^2^, CELLTREAT, StemCell Technologies, Canada) were coated with 100 μL of 1 mg/mL poly-d-lysine (Sigma-Aldrich, CH) solution on the basal side for 5 min at ambient temperature, followed by rinsing with sterile water and air-drying for 2 h. To assemble the basal layer, 100 μL of MRC-5 suspension (1 × 10^5^ cells) were seeded onto the inverted insert. After 12 h adhesion at 37 °C and 5% CO_2_, inserts were flipped down and transferred into new 12-well plates with 1 mL of fresh MEM medium. Follow a 24 h incubation to allow the MRC-5 layers to reach 90% confluency. A549 suspension was seeded onto the apical side at a density of 2 × 10^5^ cells/insert. The cultures were maintained under submerged conditions for 48 h to ensure confluence. On Day 1, the apical medium was removed to establish the ALI, and the model was maintained for 14 days with basal medium replaced every other day. On day 15, 7 × 10^4^ differentiated THP-1 cells were added onto the apical A549 layers (approximate ratio of 10:1 A549:THP-1) and allowed to integrate overnight prior to exposure.

### TEER Measurement

TEER measurement is used to access the epithelial cell barrier integrity in ALI tricultures. On day 1, TEER was determined by applying an AC square wave current of ±20 μA amplitude at 12.5 Hz, and the corresponding voltage deflection was measured by manually placing a silver/silver-chloride electrode (STX4 EVOM, World Precision Instruments, UK) at 37 °C in DMEM medium (0.5 mL of medium in upper compartment and 1.5 mL of medium in lower compartment) using a four-point galvanostatic electrochemical impedance system (GEIS) (SP-300 potentiostat, BioLogic, France). TEER measurements were obtained at regular 2 d intervals, with each data point being a mean of ten resistance measurements taken per well. Three wells were measured for each condition at each time point. Resistance values of two blank inserts without cells were averaged, and the measurements were corrected by subtracting this value. The absolute TEER values were calculated by the following equations

$$R_{tissue}=R_{total}-R_{blank}$$

$$TEER_{reported}=R_{tissue}\times M_{area}$$

where *R*_total_ is the total measured resistance (Ω) of the cell-seeded insert, including both tissue and background contributions, and *R*_blank_ is the average resistance (Ω) of the blank inserts without cells. *R*_tissue_ represents the resistance (Ω) attributable to the cellular layer. *M*_area_ is the membrane area (cm^2^) of the cell culture insert, which is used to normalize the resistance, yielding the reported TEER value in Ω·cm^2^.

### FITC-Dextran Trans-Epithelial Permeability Assay

Fluorescein isothiocyanate-labeled dextran (4 kDa; Sigma-Aldrich, CH) was dissolved in HBSS to a final concentration of 1 mg/mL. The Transwell inserts were transferred to a new 12-well plate containing 800 μL of phenol red-free MEM in the basolateral compartment. The apical medium was then aspirated and replaced with 250 μL of FITC-dextran suspension. The models were incubated for 20 min at room temperature (RT) and protected from light. Following incubation, 100 μL aliquots were collected from the basolateral medium and transferred in triplicate to Corning black-walled, clear-bottom 96-well polystyrene microplates. Fluorescence intensity was measured at λ_ex_/λ_em_ = 494/520 nm using Infinite M Nano^+^ microplate reader (Tecan, CH). The permeability was calculated as a percentage of the total tracer leaked into the basolateral compartment compared with positive control (blank insert) after subtracting the background signal of the phenol red-free MEM (blank control), the percentage of leakage was determined as follows

$$\%\;of\;positive=\frac{F_{tissue}-F_{blank}}{F_{positive}-F_{blank}}\times 100\%$$

where *F*_tissue_, *F*_blank_, and *F*_positive_ represent the fluorescence intensity measured from the 3D triculture samples, phenol red-free MEM and blank Transwell inserts, respectively.

### Endotoxin Quantification

Endotoxin concentrations in all MXene and graphene suspensions were quantified using the Pyrochrome Chromogenic Endotoxin Testing Reagents (Cape Cod Europe GmbH, DE) according to the manufacturer’s instructions.^77^ To prevent potential assay interference from the nanomaterials, samples were tested at two distinct dilutions (10- and 100-fold) and cross-referenced for consistency. Briefly, stock MXene solutions were diluted 10- and100-fold seperately in sterile, cold PBS prior to analysis (Supporting Information Figure 2a,b). For the biological exposure studies (LPS-induced acute lung injury and TGF-β-induced fibrosis models), we assessed the actual exposure medium to confirm endotoxin-free conditions throughout the assays. Aliquots of the Ta_4_C_3_-containing culture medium (DMEM/RPMI 1640, 1:1 v/v) were collected immediately upon application (0 h) and again as harvested supernatants after the 48 h incubation. These samples were then diluted in sterile cold PBS prior to testing (Supporting Information Figure 2c,d). Samples with endotoxin levels below the detection limit of 0.5 EU/mL were considered to be free of significant endotoxin contamination.

### Systematic Evaluation of Diverse 2D Materials

A549 cells were seeded in 12-well plates at a density of 1 × 10^5^ cells/well. Cell numbers were determined by using a Countess 3 Automated Cell Counter (Invitrogen, Switzerland). To simulate acute lung injury, cells were exposed to lipopolysaccharide (LPS; from*Escherichia coli*, strain O127:B8; Sigma-Aldrich, CH) at 10 μg/mL for 12 h. LPS was prepared as a 5 mg/mL stock in sterile water and stored at −20 °C. Following LPS exposure, the medium was removed and cells were treated with 20 μg/mL Ti_3_C_2_, V_2_C, Nb_2_C, Mo_2_C, Ta_4_C_3_, graphene methyl-2-pyrrolidone (NMP), or graphene oxide dispersion for 48 h. Supernatants were collected subsequently for ELISA analysis.

### Cytotoxicity In Vitro

Because the intrinsic light-absorption properties of black MXene can artificially inflate absorbance values in tetrazolium-based assays (e.g., CCK-8), cell viability was instead determined via the Calcein-AM fluorescence assay (Invitrogen, CH). A549 and MRC-5 were seeded at a density of 1.2 × 10^4^ cells/well in a 96-well plate and allowed to adhere for 24 h at 37 °C and 5% CO_2_. The cells were then exposed to 100 μL of size-fractionated MXenes at concentrations ranging from 62.5 to 800 μg/mL (62.5, 125, 250, 375, 500, 625, 800 μg/mL) for 48 h. Controls were incubated in a culture medium without MXenes. After that, the cells were washed with PBS and replaced with a Hanks Balanced Salt Solution (HBSS, Gibco, CH) containing 0.2% calcein-AM (1 μg/mL, Invitrogen, CH) for 1 h at RT, protected from light. The supernatant was removed and rinsed with HBSS three times. The fluorescence intensity was measured at λ_ex_/λ_em_ = 494/517 nm using Infinite M Nano^+^ microplate reader (Tecan, CH). Viability was calculated as a percentage relative to the BSA-treated group

$$cell\;viability=\frac{IF_{i}}{IF_{c}}\times 100\%$$

where IF_i_ and IF_c_ represent the mean fluorescence intensities of the experimental and control groups, respectively. Half-maximal inhibitory concentrations IC_50_ were derived via nonlinear regression analysis of the dose–response curves. All conditions were tested in a minimum of five biological replicates (*n* = 5).

### Immunofluorescence Staining

Prior to immunofluorescence staining, tricultures were fixed for 15 min in 4% precold paraformaldehyde (PFA) at RT, subsequently rinsed with PBS for three times, then permeabilized with 0.2% Triton X-100 for 5 min and blocked with 1% bovine serum albumin (BSA) for 1 h, both at RT. Cells were then labeled with Rhodamine Phallodin (F-actin cytoskeleton, dilution 1:1000, Abcam, CH), monoclonal mouse anti-ZO-1 Alexa Fluor 647 antibody (tight junction, dilution 1:100, Invitrogen, CH), monoclonal rabbit anti-ki67 antibody (cell proliferation, dilution 1:100, Invitrogen, CH), polyclonal rabbit antiprosurfactant protein C antibody (p-SPC, dilution 1:1000, Abcam, CH), monoclonal rabbit anti-CD11b antibody (dilution 1:500, Abcam, CH), polyclonal rabbit anti-CD86 antibody (dilution 1:1000, Invitrogen, CH), and monoclonal rabbit anti-CD206 antibody (dilution 1:1000, Abcam, CH) diluted in 1% BSA for 2 h at RT. For SPC, cells were incubated in the goat antirabbit Alexa Fluor 488 antibody (Abcam, CH) diluted in 1:1000 for 1 h at RT. Nuclei was stained with 4′,6-diamidino-2-phenylindole (DAPI, dilution 1:5000, Invitrogen, CH) for 10 min. After the PBS washing steps, inserts were cut off by scrapers and mounted onto the coverslips. Cell morphologies were visualized via an inverted spinning disk confocal microscope (SDCM, Nikon, DE) equipped with an NIS element software package. Image processing was conducted with ImageJ.

### Ta_4_C_3_ Treatment in a 3D Triculture Model of Acute Lung Injury

To simulate acute lung injury, on day 16, the apical surface of the 3D model was treated with 200 μL of 10 μg/mL LPS in DMEM/RPMI 1640 (1:1, v/v) medium for 12 h under submerged conditions. On day 17, the apical medium was removed, and 200 μL of size-fractionated Ta_4_C_3_ (50 μg/mL) was administered apically. TAK-242 (1 μM; Sigma-Aldrich, CH) was utilized as a positive therapeutic control, while tissues treated only with BSA served as the negative controls. This inoculation method mimics liquid layer deposition on the air-facing epithelium, consistent with clinical inhalation delivery. On day 19, the basolateral medium was collected and centrifuged at 15,000 rpm for 10 min to pellet the Ta_4_C_3_ particles and any cellular debris. Medium was centrifuged at 15,000 rpm for 10 min to remove any cell debris. IL-6 and IL-8 release was accessed via ELISA, using the commercially available single-wash 90 min SimpleStep ELISA kit (Abcam, CH), according to the manufacturer’s protocol. Because 2D nanomaterials can potentially interfere with optical assays, we conducted interference controls to ensure Ta_4_C_3_ did not quench the dyes or adsorb the analytes. Specifically, 50 μg/mL Ta_4_C_3_ was mixed with the protein standards for IL-6 and IL-8 and incubated. The mixture was then centrifuged at 15,000 rpm for 10 min to pellet the Ta_4_C_3_ particles, and the resulting supernatant was measured. These spiked samples were compared against Ta_4_C_3_-free protein standards; the resulting standard curves (Supporting Information Figure S12) showed no significant difference in absorbance or linear slopes, confirming that the flakes did not sequester the cytokines or interfere with the detection system.

### Intracellular ROS Assessment

Intracellular ROS generation was assessed using the DCFDA/H_2_DCFDA Cellular ROS Assay Kit (Abcam, CH). The assay utilizes the cell-permeant probe DCFH-DA, which passively diffuses into cells and is deacetylated by intracellular esterases to nonfluorescent H_2_DCF, resulting in its intracellular entrapment. Upon oxidation by ROS, H_2_DCF is converted to highly fluorescent 2′,7′-dichlorofluorescein (DCF), with fluorescence intensity directly proportional to oxidative stress levels. Briefly, the stock solution was diluted to a working concentration of 20 μM, and the apical compartments were incubated with 500 μL of the probe for 45 min at 37 °C in the dark. Following the loading step, cells were washed three times with dilution buffer to remove noninternalized dye and residual MXene. BSA-only treated models served as the negative control, while MXene-exposed models processed without the DCFH-DA probe were included as background controls to correct for intrinsic material autofluorescence. Intracellular fluorescence distributions were visualized by using a spinning disk confocal microscope (SDCM, Nikon, DE).

### Ta_4_C_3_ Treatment in a 3D Triculture Model of Pulmonary Fibrosis

To establish a 3D model of pulmonary fibrosis, tricultures were stimulated with transforming growth factor-beta 1 (TGF-β; PeproTech, USA). A 100 μg/mL stock solution was prepared in 10 mM citric acid (pH 3.0) and stored at −20 °C. At day 16, the 3D models were treated both apically (200 μL) and basolaterally (1 mL) with 50 ng/mL TGF-β for 24 h. On day 17, size-fractionated Ta_4_C_3_ was administered to both the apical and basolateral compartments at concentrations of 200 μg/mL in DMEM/RPMI 1640 (1:1, v/v) medium for a 48 h exposure period. Tranilast (10 mM; Abcam, Switzerland), an antifibrotic agent, served as the positive therapeutic control, while tissues treated only with BSA were utilized as negative controls. On day 19, the basolateral medium was collected and centrifuged at 15,000 rpm for 10 min to pellet the Ta_4_C_3_ particles and any cellular debris. The secretion of fibronectin and pro-collagen I-α1 into the basolateral medium was quantified using DuoSet ELISA kits (R&D Systems, CH) according to the manufacturer’s protocol. Interference controls were performed to ensure that Ta_4_C_3_ did not quench the dyes or adsorb the analytes. Specifically, 200 μg/mL Ta_4_C_3_ was mixed with the protein standards for fibronectin and pro-collagen I-α1 and incubated. The mixture was then centrifuged at 15,000 rpm for 10 min to pellet the Ta_4_C_3_ particles, and the resulting supernatant was measured. These spiked samples were compared against Ta_4_C_3_-free protein standards; the resulting standard curves (Supporting Information Figure 12) showed no significant difference in absorbance or linear slopes, confirming that the flakes did not sequester the cytokines or interfere with the detection system.

### TEM Analysis for the Cellular Uptake of Ta_4_C_3_

Sample processing of A549 epithelial cells exposed to Ta_4_C_3_ into resin was performed in a PELCO BioWave, Pro+ microwave system (Ted Pella Inc., USA) for imaging morphology. The samples were washed three times with cold PBS to remove loosely adherent particles, followed by fixation in 4% PFA and 2.5% glutaraldehyde in 0.1 M PBS buffer at pH 7.3. Cells were then scraped and pelleted, encapsulated in 3% agarose followed by postfixation in 1% osmium tetroxide (OsO_4_) in 0.1 M PBS. After washing once in PBS and twice in distilled water (dH_2_O), further postfixation was performed in 1% tannic acid for 20 min on ice, washed twice in dH_2_O, immersed in 0.5% uranyl acetate in dH_2_O, and washed three times in dH_2_O. Dehydration was accomplished in a graded series of ethanol (25%, 50%, 75%, 95%, and 100%) followed by 100% acetone. Dehydrated samples were then infiltrated in a graded acetone-Epon (Electron Microscopy Sciences, Hatfield, PA) series at 30%, 50%, 70% and finally at 100% Epon resin. Polymerization of Epon resin was achieved at 60 °C for 72 h in the resin molds. Ultrathin sections of 60 nm were obtained with a diamond knife (Diatome Ltd., CH) using a Leica UC7 ultramicrotome (Leica Microsystems, CH), placed on Formvar/carbon coated TEM grids (Quantifoil, DE), and stained with 2% aqueous uranyl acetate and Reynold’s lead citrate. Micrographs of the stained sections were imaged using the Thermo Fisher Scientific (TFS) Talos L120C TEM (Thermo Fisher Scientific, USA) equipped with a BM-Ceta CMOS camera operating at a 120 kV acceleration voltage in the bright field mode.

### EDX Analysis

A549 epithelial cells exposed to Ta_4_C_3_ used for EDX analysis were from the same stained sections used for imaging the morphology. Ta_4_C_3_ MXene samples were prepared for EDX by deposition of 5 μL of suspension onto glow discharged TEM grids (Quantifoil, DE) for 30 s and rinsed with distilled water. EDX analysis was performed with the TFS Talos F200X TEM with a Super-X EDS system with a 4-detection configuration (Thermo Fisher Scientific, USA). Data acquisition and EDX evaluation were accomplished using TFS Velox software (Thermo Fisher Scientific, USA).

### Statistical Analysis

Statistical analysis was performed using GraphPad Prism 9 software (GraphPad Software Inc., CA, USA). For each data point, three independent experiments were performed, and all data are presented as the mean ± standard deviation. *t*-tests were performed, and results were considered significant if *p* < 0.05. NS indicates no significance.

## Supplementary Material



## Notes

Detailed experimental procedures for the synthesis of Ti_3_C_2_, Nb_2_C, Mo_2_C, V_2_C, graphene *N*-NMP, and graphene oxide dispersions; protocols for 2D submerged cell culture and 3D ALI multilayered tricultures with Ta_4_C_3_ treatment; TEER profiles in 3D ALI monocultures; endotoxin content analysis for all tested materials; dose-dependent cytotoxicity profiles in A549 and MRC-5 cells; cellular internalization and STEM–EDX elemental analysis; comparative anti-inflammatory efficacy in 2D submerged vs 3D ALI cocultures; morphological assessment of the 3D ALI coculture under pro-inflammatory stimulation; antioxidant efficacy evaluation; subtherapeutic and dose-dependent antifibrotic evaluations; morphological changes under pro-fibrotic stimulation; epithelial-fibroblast cross-talk; and interference controls in ELISA assay (PDF)

## References

[1] Jin Z., Gao Q., Wu K., Ouyang J., Guo W., Liang X. J. (2023). Harnessing
inhaled nanoparticles to overcome the pulmonary barrier for respiratory
disease therapy. Adv. Drug Deliv. Rev..

[2] Ahmad J., Akhter S., Ahmad J., Rizwanullah M., Rahman M., Zaki Ahmad M., Rizvi M. M. A., Ahmad F. J., Amin S., Kamal M. A. (2015). Nanotechnology-based inhalation treatments
for lung cancer: state of the art. Nanotechnol.,
Sci. Appl..

[3] Zhang S., Li R., Jiang T., Gao Y., Zhong K., Cheng H., Chen X., Li S. (2024). Inhalable
nanomedicine for lung cancer
treatment. Smart Mater. Med..

[4] Yang W., Peters J. I., Williams R. O. (2008). Inhaled nanoparticles--a
current review. Int. J. Pharm..

[5] Poulakou G., Matthaiou D. K., Nicolau D. P., Siakallis G., Dimopoulos G. (2017). Inhaled Antimicrobials
for Ventilator-Associated Pneumonia:
Practical Aspects. Drugs.

[6] Hillman T., Mortimer F., Hopkinson N. S. (2013). Inhaled
drugs and global warming:
time to shift to dry powder inhalers. BMJ.

[7] Zhang C., D’Angelo D., Buttini F., Yang M. (2024). Long-acting inhaled
medicines: Present and future. Adv. Drug Deliv.
Rev..

[8] Wu W., Ge H., Zhang L., Lei X., Yang Y., Fu Y., Feng H. (2020). Evaluating the Cytotoxicity
of Ti(3)­C(2) MXene to Neural Stem Cells. Chem.
Res. Toxicol..

[9] Lim G. P., Soon C. F., Jastrzębska A.
M., Ma N. L., Wojciechowska A. R., Szuplewska A., Wan Omar W. I., Morsin M., Nayan N., Tee K. S. (2021). Synthesis, characterization and biophysical
evaluation of the 2D Ti2CTx MXene using 3D spheroid-type cultures. Ceram. Int..

[10] Liu J., Lu W., Lu X., Zhang L., Dong H., Li Y. (2022). Versatile
Ti­(3)­C­(2)­T (x) MXene for free-radical scavenging. Nano Res..

[11] Ozulumba T., Ingavle G., Gogotsi Y., Sandeman S. (2021). Moderating cellular
inflammation using 2-dimensional titanium carbide MXene and graphene
variants. Biomater. Sci..

[12] Fusco L., Gazzi A., Shuck C. E., Orecchioni M., Ahmed E. I., Giro L., Zavan B., Yilmazer A., Ley K., Bedognetti D. (2023). V­(4) C(3) MXene Immune Profiling and Modulation
of T Cell-Dendritic Cell Function and Interaction. Small Methods.

[13] Hou L., Gong F., Liu B., Yang X., Chen L., Li G., Gong Y., Liang C., Yang N., Shen X. (2022). Orally administered titanium carbide nanosheets as anti-inflammatory
therapy for colitis. Theranostics.

[14] Khajeh-Hosseini-Dalasm N., Longest P. W. (2015). Deposition
of Particles in the Alveolar Airways: Inhalation
and Breath-Hold with Pharmaceutical Aerosols. J. Aerosol Sci..

[15] Zhang B., Wu Y., Bai W., Aaron D. (2024). Franklin. MXene-Contacted Carbon
Nanotube Thin-Film Transistors Using Aerosol Jet Printing. IEEE Trans. Mater. Electron Device.

[16] Andrews J. P. M., Joshi S. S., Tzolos E., Syed M. B., Cuthbert H., Crica L. E., Lozano N., Okwelogu E., Raftis J. B., Bruce L. (2024). First-in-human controlled
inhalation of thin graphene
oxide nanosheets to study acute cardiorespiratory responses. Nat. Nanotechnol..

[17] Nelson C. P., Brown P., Fitzpatrick S., Ford K. A., Howard P. C., MacGill T., Margerrison E. E. C., O’Shaughnessy J., Patterson T. A., Raghuwanshi R. (2024). Advancing alternative
methods to reduce animal testing. Science.

[18] U.S. Food and Drug Administration . FDA’s Predictive Toxicology Roadmap. https://www.regulations.gov/docket/FDA-2025-D-6131 (accessed March 22, 2026).

[19] Rahman, S. , Shadiqur Rashid Roni, M. ; Tariq, I. ; Ismaiel, O. ; Ford, K. A Microphysiological Model of Human Lung Airway for Evaluating Dissolution and Permeability of Inhaled Drugs. In 2023 FDA Science Forum, 2023.

[20] Teimouri, A. ; Yeung, P. ; Agu, R. 2D vs. 3D Cell Culture Models for in Vitro Topical (Dermatological) Medication Testing; Intechopen, 2019

[21] Hittinger M., Schneider-Daum N., Lehr C. M. (2017). Cell and tissue-based in vitro models
for improving the development of oral inhalation drug products. Eur. J. Pharm. Biopharm..

[22] Poornima K., Francis A. P., Hoda M., Eladl M. A., Subramanian S., Veeraraghavan V. P., El-Sherbiny M., Asseri S. M., Hussamuldin A. B. A., Surapaneni K. M. (2022). Implications of Three-Dimensional
Cell Culture in Cancer Therapeutic Research. Front. Oncol..

[23] Lacroix G., Koch W., Ritter D., Gutleb A. C., Larsen S. T., Loret T., Zanetti F., Constant S., Chortarea S., Rothen-Rutishauser B. (2018). Air-Liquid Interface In Vitro Models for
Respiratory Toxicology Research: Consensus Workshop and Recommendations. Appl. In Vitro Toxicol..

[24] Barosova H., Karakocak B. B., Septiadi D., Petri-Fink A., Stone V., Rothen-Rutishauser B. (2020). An In Vitro Lung System to Assess
the Proinflammatory Hazard of Carbon Nanotube Aerosols. Int. J. Mol. Sci..

[25] Silva S., Bicker J., Falcao A., Fortuna A. (2023). Air-liquid interface
(ALI) impact on different respiratory cell cultures. Eur. J. Pharm. Biopharm..

[26] Kryvenko V., Vadasz I. (2024). Modeling the Human Alveolar Epithelium:
Promises and
Challenges. Am. J. Respir. Cell Mol. Biol..

[27] Araya J., Cambier S., Morris A., Finkbeiner W., Nishimura S. L. (2006). Integrin-mediated transforming growth
factor-beta activation
regulates homeostasis of the pulmonary epithelial-mesenchymal trophic
unit. Am. J. Pathol..

[28] Movia D., Bazou D., Prina-Mello A. (2019). ALI multilayered
co-cultures mimic
biochemical mechanisms of the cancer cell-fibroblast cross-talk involved
in NSCLC MultiDrug Resistance. BMC Cancer.

[29] Nalayanda D. D., Puleo C., Fulton W. B., Sharpe L. M., Wang T. H., Abdullah F. (2009). An open-access microfluidic
model for lung-specific
functional studies at an air-liquid interface. Biomed. Microdevices.

[30] Walter F. R., Valkai S., Kincses A., Petneházi A., Czeller T., Veszelka S., Ormos P., Deli M. A., Dér A. (2016). A versatile lab-on-a-chip tool for modeling biological
barriers. Sens. Actuators, B.

[31] Cooper J. R., Abdullatif M. B., Burnett E. C., Kempsell K. E., Conforti F., Tolley H., Collins J. E., Davies D. E. (2016). Long Term
Culture
of the A549 Cancer Cell Line Promotes Multilamellar Body Formation
and Differentiation towards an Alveolar Type II Pneumocyte Phenotype. PLoS One.

[32] Ramezani
Farani M., Mirzaee D., Hatami A., Kumar K., Ghoreishian S. M., Huh Y. S. (2026). Biocompatibility and immunomodulation
of MXenes for targeted delivery of bioactive agents and drugs. Bioact. Mater..

[33] Fusco L., Gazzi A., Shuck C. E., Orecchioni M., Alberti D., D’Almeida S. M., Rinchai D., Ahmed E., Elhanani O., Rauner M. (2022). Immune Profiling and
Multiplexed Label-Free Detection of 2D MXenes by Mass Cytometry and
High-Dimensional Imaging. Adv. Mater..

[34] Jastrzebska A. M., Scheibe B., Szuplewska A., Rozmyslowska-Wojciechowska A., Chudy M., Aparicio C., Scheibe M., Janica I., Ciesielski A., Otyepka M. (2021). On the rapid in situ
oxidation of two-dimensional V(2)­CT­(z) MXene in culture cell media
and their cytotoxicity. Mater. Sci. Eng., C.

[35] Jang J. H., Lee E. J. (2021). Influence of MXene Particles with
a Stacked-Lamellar
Structure on Osteogenic Differentiation of Human Mesenchymal Stem
Cells. Materials.

[36] Jastrzebska A. M., Szuplewska A., Wojciechowski T., Chudy M., Ziemkowska W., Chlubny L., Rozmyslowska A., Olszyna A. (2017). In vitro studies on
cytotoxicity of delaminated Ti(3)­C(2) MXene. J. Hazard. Mater..

[37] Han J., Kim Y. S., Lim M. Y., Kim H. Y., Kong S., Kang M., Choo Y. W., Jun J. H., Ryu S., Jeong H. Y. (2018). Dual
Roles of Graphene Oxide To Attenuate Inflammation
and Elicit Timely Polarization of Macrophage Phenotypes for Cardiac
Repair. ACS Nano.

[38] Li M. F., Xu L., Guo M. D., Shang H., Luo X., Ma Y. A. (2025). Recent
Advances in Tantalum Carbide MXenes: Synthesis, Structure, Properties,
and Novel Applications. Crystals.

[39] Rafieerad A., Amiri A., Sequiera G. L., Yan W., Chen Y., Polycarpou A. A., Dhingra S. (2021). Development of Fluorine-Free Tantalum
Carbide MXene Hybrid Structure as a Biocompatible Material for Supercapacitor
Electrodes. Adv. Funct. Mater..

[40] Adomaviciute-Grabusove S., Popov A., Ramanavicius S., Sablinskas V., Shevchuk K., Gogotsi O., Baginskiy I., Gogotsi Y., Ramanavicius A. (2024). Monitoring Ti(3)­C(2)­T­(x) MXene Degradation
Pathways Using Raman Spectroscopy. ACS Nano.

[41] ISO . Biological Evaluation of Medical Devices—Part 5: Tests for In Vitro Cytotoxicity, ISO 10993-5:2009; Geneva, 2009

[42] Radiom M., Sarkis M., Brookes O., Oikonomou E. K., Baeza-Squiban A., Berret J. F. (2020). Pulmonary surfactant inhibition of
nanoparticle uptake by alveolar epithelial cells. Sci. Rep..

[43] Heyder J. (2004). Deposition
of inhaled particles in the human respiratory tract and consequences
for regional targeting in respiratory drug delivery. Proc. Am. Thorac. Soc..

[44] Rejman J., Oberle V., Zuhorn I. S., Hoekstra D. (2004). Size-dependent
internalization
of particles via the pathways of clathrin- and caveolae-mediated endocytosis. Biochem. J..

[45] Klionsky D. J., Eskelinen E. L., Deretic V. (2014). Autophagosomes, phagosomes, autolysosomes,
phagolysosomes, autophagolysosomes··· wait, I’m
confused. Autophagy.

[46] Hollenstein D. M., Kraft C. (2020). Autophagosomes are formed at a distinct cellular structure. Curr. Opin. Cell Biol..

[47] Dutra
Silva J., Su Y., Calfee C. S., Delucchi K. L., Weiss D., McAuley D. F., O’Kane C., Krasnodembskaya A. D. (2021). Mesenchymal stromal cell extracellular vesicles rescue
mitochondrial dysfunction and improve barrier integrity in clinically
relevant models of ARDS. Eur. Respir. J..

[48] He Y., Peng E., Ba X., Wu J., Deng W., Huang Q., Tong Y., Shang H., Zhong Z., Liu X. (2025). ROS Responsive Cerium
Oxide Biomimetic Nanoparticles
Alleviates Calcium Oxalate Crystals Induced Kidney Injury via Suppressing
Oxidative Stress and M1Macrophage Polarization. Small.

[49] Deng J., Xian D., Cai X., Liao S., Lei S., Han F., An Y., He Q., Quan G., Wu C. (2023). Surface-Engineered Vanadium Carbide MXenzyme for Anti-Inflammation
and Photoenhanced Antitumor Therapy of Colon Diseases. Adv. Funct. Mater..

[50] Shi H., Guo Y., Geng C., Chen L., He S., He W., Chen Y., Chan Y. K., Wang C., Deng Y. (2025). Engineered
Bio-Heterojunction with Robust ROS-Scavenging and Anti-Inflammation
for Targeted Acute Pancreatitis Therapy. Adv.
Funct. Mater..

[51] Klein S. G., Serchi T., Hoffmann L., Blomeke B., Gutleb A. C. (2013). An improved
3D tetraculture system mimicking the cellular organisation at the
alveolar barrier to study the potential toxic effects of particles
on the lung. Part. Fibre Toxicol..

[52] Baxter S. K., Irizarry-Caro R. A., Vander Heiden J. A., Arron J. R. (2025). Breaking the cycle:
should we target inflammation, fibrosis, or both?. Front. Immunol..

[53] Wilson M. S., Wynn T. A. (2009). Pulmonary fibrosis:
pathogenesis, etiology and regulation. Mucosal
Immunol..

[54] King T. E., Pardo A., Selman M. (2011). Idiopathic
pulmonary fibrosis. Lancet.

[55] Yue X., Shan B., Lasky J. A. (2010). TGF-beta:
Titan of Lung Fibrogenesis. Curr. Enzyme Inhib..

[56] Ge Z., Chen Y., Ma L., Hu F., Xie L. (2024). Macrophage
polarization and its impact on idiopathic pulmonary fibrosis. Front. Immunol..

[57] Ueha S., Shand F. H., Matsushima K. (2012). Cellular and
molecular mechanisms
of chronic inflammation-associated organ fibrosis. Front. Immunol..

[58] Park S., Park M., Kim B. H., Lee J. E., Park H. J., Lee S. H., Park C. G., Kim M. H., Kim R., Kim E. H. (2015). Acute
suppression of TGF-ss with local, sustained release
of tranilast against the formation of fibrous capsules around silicone
implants. J. Controlled Release.

[59] Wang X., Xia T., Addo Ntim S., Ji Z., Lin S., Meng H., Chung C. H., George S., Zhang H., Wang M. (2011). Dispersal state of multiwalled
carbon nanotubes elicits profibrogenic
cellular responses that correlate with fibrogenesis biomarkers and
fibrosis in the murine lung. ACS Nano.

[60] Card J. W., Zeldin D. C., Bonner J. C., Nestmann E. R. (2008). Pulmonary applications
and toxicity of engineered nanoparticles. Am.
J. Physiol. Lung Cell Mol. Physiol..

[61] Lim G. P., Soon C. F., Ma N. L., Morsin M., Nayan N., Ahmad M. K., Tee K. S. (2021). Cytotoxicity of
MXene-based nanomaterials
for biomedical applications: A mini review. Environ. Res..

[62] Alhabeb M., Maleski K., Anasori B., Lelyukh P., Clark L., Sin S., Gogotsi Y. (2017). Guidelines
for Synthesis and Processing of Two-Dimensional
Titanium Carbide (Ti3C2Tx MXene). Chem. Mater..

[63] Aleksandrova M., Kurtev N., Pandiev I. (2024). Effect of
MXene nanosheet sticking
on supercapacitor device performance. Appl.
Sci..

[64] Zhang C. J., Pinilla S., McEvoy N., Cullen C. P., Anasori B., Long E., Park S.-H., Seral-Ascaso A., Shmeliov A., Krishnan D. (2017). Oxidation
Stability
of Colloidal Two-Dimensional Titanium Carbides (MXenes). Chem. Mater..

[65] Rothen-Rutishauser B. M., Kiama S. G., Gehr P. (2005). A three-dimensional
cellular model
of the human respiratory tract to study the interaction with particles. Am. J. Respir. Cell Mol. Biol..

[66] Kuhn D. A., Vanhecke D., Michen B., Blank F., Gehr P., Petri-Fink A., Rothen-Rutishauser B. (2014). Different
endocytotic uptake mechanisms
for nanoparticles in epithelial cells and macrophages. Beilstein J. Nanotechnol..

[67] Mizushima N., Komatsu M. (2011). Autophagy: renovation of cells and tissues. Cell.

[68] Zhu L., Zhu D., Ran J., Li M., Lai Z., Zhou Y., Luo L., Liu X., Mao K., Tian K. (2024). Autophagy aggravates
multi-walled carbon nanotube-induced ferroptosis by suppressing PGC-1
dependent-mitochondrial biogenesis in lung epithelial cells. Chem. Biol. Interact..

[69] Ouhajji S., Swart E., Volker D., Schwirn K., Fadeel B., Oomen A. G. (2025). Titanium carbide
MXenes - Early identification of safety,
sustainability and regulatory issues. NanoImpact.

[70] OECD . Test No. 436: Acute Inhalation Toxicity-Acute Toxic Class Method: Paris, 2009.

[71] International Organization for Standardization . Nanotechnologies-Guidance on the Measurement of Nanoparticle Number Concentration, ISO/TS 24672:2023; International Organization for Standardization: Geneva, Switzerland, 2023, https://www.iso.org/standard/79369.html.

[72] Filippov S. K., Khusnutdinov R., Murmiliuk A., Inam W., Zakharova L. Y., Zhang H., Khutoryanskiy V. V. (2023). Dynamic light scattering and transmission
electron microscopy in drug delivery: a roadmap for correct characterization
of nanoparticles and interpretation of results. Mater. Horiz..

[73] Barosova H., Meldrum K., Karakocak B. B., Balog S., Doak S. H., Petri-Fink A., Clift M. J. D., Rothen-Rutishauser B. (2021). Inter-laboratory
variability of A549 epithelial cells grown under submerged and air-liquid
interface conditions. Toxicol. In Vitro.

[74] Michelini S., Mawas S., Kuresepi E., Barbero F., Simunovic K., Miremont D., Devineau S., Schicht M., Ganin V., Haugen O. P. (2025). Pulmonary hazards of
nanoplastic particles:
a study using polystyrene in in vitro models of the alveolar and bronchial
epithelium. J. Nanobiotechnology.

[75] Gillich A., Zhang F., Farmer C. G., Travaglini K. J., Tan S. Y., Gu M., Zhou B., Feinstein J. A., Krasnow M. A., Metzger R. J. (2020). Capillary cell-type specialization
in the alveolus. Nature.

[76] Movia D., Bazou D., Volkov Y., Prina-Mello A. (2018). Multilayered
Cultures of NSCLC cells grown at the Air-Liquid Interface allow the
efficacy testing of inhaled anti-cancer drugs. Sci. Rep..

[77] Agarwal V., Yue Y., Zhang X., Feng X., Tao Y., Wang J. (2023). Spatial and
temporal distribution of endotoxins, antibiotic resistance genes and
mobile genetic elements in the air of a dairy farm in Germany. Environ. Pollut..

