# Supplementary material for: Predictive Screening of Ta4C3 MXene as an Inhalable Nanotherapeutic Based on an Advanced 3D Air–Liquid Interface Lung Model
Source: ACS Nano. 2026 Apr 2;20(14):11466–83. doi: 10.1021/acsnano.6c03602 (PMC13085849; doi:10.1021/acsnano.6c03602)
Supplement: Supplementary file 1 [file nn6c03602_si_001.pdf]

Predictive Screening of Ta_4C_3 MXene as an Inhalable Nanotherapeutic Based on an Advanced 3D Air-Liquid Interface Lung Model

Ying Kong,[†,#] Nicole J. Machi,[‡] Fuze Jiang,[‖] Eszter J. Barthazy Meier,[‡] Viktoria Agarwal,[†,#] Zhou Dong,[⊥] Sung Sik Lee[*,‡,§] and Jing Wang[*,†,#]

[†]Institute of Environmental Engineering, ETH Zürich, 8093 Zürich, Switzerland

[‡]Scientific Center for Optical and Electron Microscopy (ScopeM), ETH Zürich, 8093 Zürich, Switzerland

[§]Institute of Biochemistry, ETH Zürich, 8093 Zürich, Switzerland

[‖]Department of Information Technology and Electrical Engineering, ETH Zürich, 8092 Zürich, Switzerland

[⊥]Department of Health Sciences and Technology, ETH Zürich, 8092 Zürich, Switzerland

[#]Laboratory for Building Energy Materials and Components, Empa, Swiss Federal Laboratories for Materials Science and Technology, 8600 Zürich, Switzerland

[*]Email: sungsik.lee@scopem.ethz.ch.

[*]Email: jing.wang@ifu.baug.ethz.ch.

Materials and Methods

Synthesis of the Ti_3C_2 MXene dispersion. 2.0 g of Ti_3AlC_2 powder was slowly added to a PTFE container holding 100 mL of an aqueous solution containing 3.1 g LiF and 40 mL of 9 M HCl. The mixture was stirred for 48 h at 40–45 °C. The resulting suspension was washed with deionized (DI) water and centrifuged at 3500 rpm for 5 min repeated 7–8 times until the supernatant pH reached 6. The sediment was then re-dispersed in DI water, sonicated for 1 h under argon flow, and centrifuged at 3500 rpm for 30 min. The supernatant containing delaminated 2D sheets of Ti_3C_2 MXene was collected.

Synthesis of the Nb_2C MXene dispersion. 2.0g Nb_2AlC was slowly dispersed into 250 mL of PTFE aqueous solution containing 30 mL of 49% HF, followed by stirring for 72 h at 50 °C. A black solid precipitated slowly after repeated centrifugation at 8500 rpm for 5 min. It was mixed with 50 mL 20 wt% TMAOH for 24 h at room temperature and then centrifuged at 8500 rpm for 5 min to remove the TMAOH. Then it was sonicated for 1h and using deionized water as the dispersing medium After centrifugation at 3500 rpm for 30 min, the supernatant containing delaminated 2D sheets of Nb_2C MXene was collected.

Synthesis of the Mo_2C MXene dispersion. 2.0 g Mo_2AlC was slowly dispersed into 100 mL of PTFE aqueous solution containing 20 mL of 49% HF, followed by stirring for 120 h at 55-60 °C. A black solid precipitated slowly after 7-8 times of centrifugation at 6000 rpm for 5 min. It was mixed with 50 mL 20 wt% TMAOH for 24h at room temperature and then centrifuged at 6000 rpm for 5 min. The mixture was washed several times with ethanol and sonicated with deionized water for 1 h. After centrifugation at 3000 rpm for 15 min, the supernatant containing delaminated 2D sheets of Mo_2C MXene was collected.

Synthesis of the V_2C MXene dispersion. 2.0 g V_2AlC was slowly dispersed into 250 mL of PTFE aqueous solution containing mixture of 20 mL of 40% HF, 20 mL of HCI and 10 mL of H_2O, followed by stirring for 48 h at 40°C. A black solid precipitated slowly after 7-8 times of centrifugation at 8500 rpm for 5 min. It was mixed with 50 mL 20 wt% TMAOH for 24 h at room temperature and then centrifuged at 8500 rpm for 5 min. After centrifugation at 3500 rpm for 30 min, the supernatant containing delaminated 2D sheets of V_2C MXene was collected.

Synthesis of the graphene NMP dispersion. To prepare a stable single-layer graphene NMP dispersion, 0.2 g of polyvinylpyrrolidone (PVP, molecular weight 50,000) was dissolved in 20 mL of N-methyl-2-pyrrolidone (NMP) using ultrasonication to ensure a uniform and homogeneous solution. Subsequently, 0.001 g of expanded graphite was added to the PVP-NMP solution, and the mixture was sonicated for 40 min at room temperature to exfoliate the graphite and achieve uniform dispersion. An ice-water bath was intermittently applied during sonication to prevent overheating.

Synthesis of the graphene oxide. Graphene oxide was prepared by adding concentrated inorganic acid to a sealed reactor, followed by the addition of graphite powder under stirring. The mixture was cooled to 0–4 °C, and potassium permanganate was gradually added to initiate the reaction, which proceeded for 0.5–2 h with a material ratio of 23 mL acid and 1:4–6 graphite-to-potassium permanganate. The mixture was then heated to 60–70 °C and sealed for 12–24 h. Afterward, the oxidized graphite was transferred into a frozen hydrogen peroxide solution (distilled water to hydrogen peroxide ratio of 10:1, using 100 mL water per 1 g graphite). Once the ice melted, the mixture was stirred, vacuum-filtered, and washed with 5% HCl, distilled water, and ethanol until sulfate ions were removed and the pH was neutral. The graphene oxide was dried at 50 °C for 6 h.

To minimize oxidation and maintain material integrity for research, all materials were stored at 4 °C and used within three months to ensure optimal freshness and stability.

XRD analysis. X-ray diffraction (XRD) patterns of all the above materials were performed under the same conditions as for Ta_4C_3.

2D submerged mono-cultures. A549 cells and MRC-5 cells were seeded into 12-well plates at a density of 1×10^5 cells/well for ELISA assays, and into 96-well plates at a density of 1.2×10^4 cells/well for the calcein-AM viability test. Cell counting was performed using a Countess 3 Automated Cell Counter (Invitrogen, Switzerland). Following a 24 h incubation period, the cells formed a monolayer reaching 60–70% confluency, at which point they were used for subsequent exposure experiments.

3D ALI multilayered co-cultures. To produce the co-cultures, on day 2, MRC-5 cells were seeded onto the basolateral side of inserts coated with poly-D-Lysine. Briefly, microporous polyethylene (PET) membrane inserts (0.4 μm pore size, pore density 2×10^6 pores/cm^2, CELLTREAT, StemCell Technologies, Canada) were coated with 100 μL of 1 mg/mL poly-D-lysine (Sigma-Aldrich, CH) solution on basal side for 5 min at ambient temperature, followed by rinsing with sterile water and air-drying for 2 h. To assemble the basal layer, 100 μL of MRC-5 suspension (1×10^5 cells) were seeded onto the inverted insert. After 12 h adhesion at 37 °C and 5% CO_2, inserts were flipped down and transferred into new 12-well plates with 1 mL fresh MEM medium. Follow a 24 h incubation to allow the MRC-5 layers to reach 90% confluency. A549 suspension was seeded onto the apical side at a density of 2×10^5 cells/insert. The cultures maintained under submerged conditions for 48 h to ensure confluence. On day 1, the apical medium was removed to establish air-liquid interface (ALI), and the model was maintained for 14 days with basal medium replaced every other day before exposure.

Ta_4C_3 Treatment in acute lung injury model and pulmonary fibrosis model.

For acute lung injury model, on day 15, the apical surface of the 3D model was treated with 200 µL of 10 µg/mL LPS in DMEM medium for 12 h under submerged conditions. On day 16, the apical medium was removed, and 200 µL of size-fractionated Ta_4C_3 (50 µg/mL) was administered apically for another 48 h. The same exposure period was applied to 2D submerged cultures. Polymyxin B (10 µM; Sigma-Aldrich, CH) was utilized as a positive therapeutic control, while tissues treated only with BSA served as the negative controls.

For pulmonary fibrosis model, on day 15, the 3D models were treated both apically (200 µL) and basolaterally (1 mL) with 50 ng/mL TGF-β for 24 h. On day 16, size-fractionated Ta_4C_3 was administered to both the apical and basolateral compartments at concentrations of 200 µg/mL in DMEM medium for a 48 h exposure period. The same exposure period was applied to 2D submerged cultures. Tranilast served as the positive therapeutic control, while tissues treated only with BSA were utilized as negative controls.

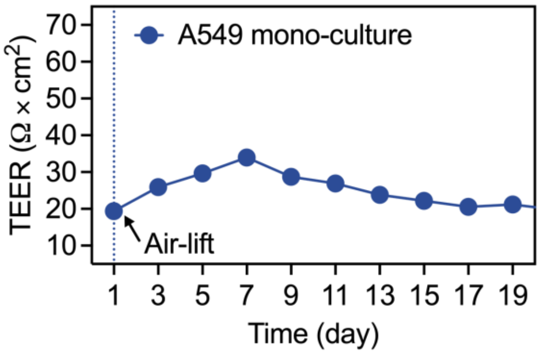

Supplementary Figure 1. TEER profiles monitored over 19 days in 3D air-liquid interface (ALI) mono-cultures.

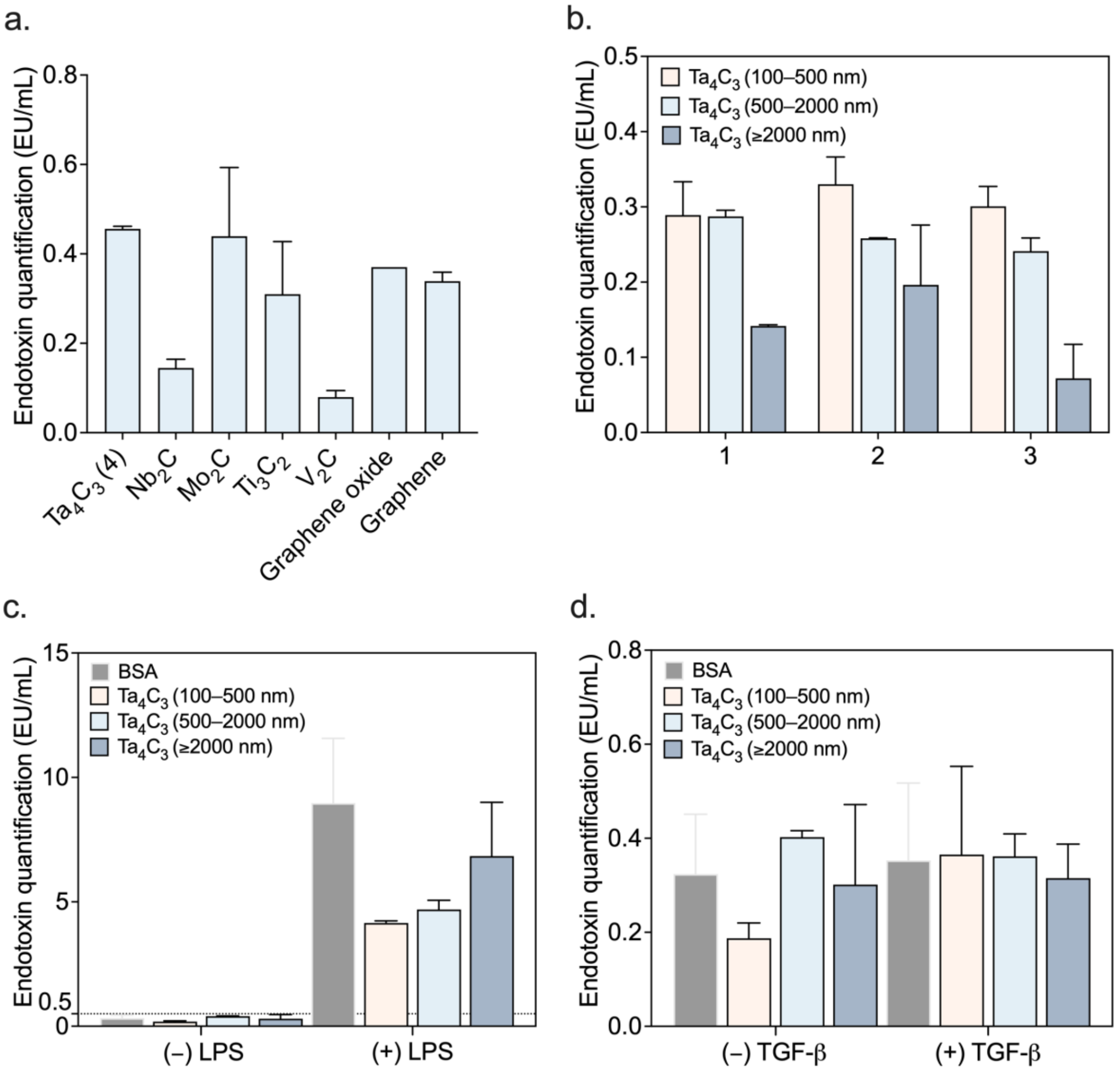

Supplementary Figure 12. (a, b) Endotoxin levels measured for all materials used in our study. Numbers indicate different individual batches (e.g., 1, 2, 3, 4). Endotoxin levels in basal medium collected before and after 48h of Ta_4C_3 treatment in the 3D ALI tri-culture model, under the LPS-induced acute lung injury model (c) and the TGF-β-induced lung fibrosis model (d).

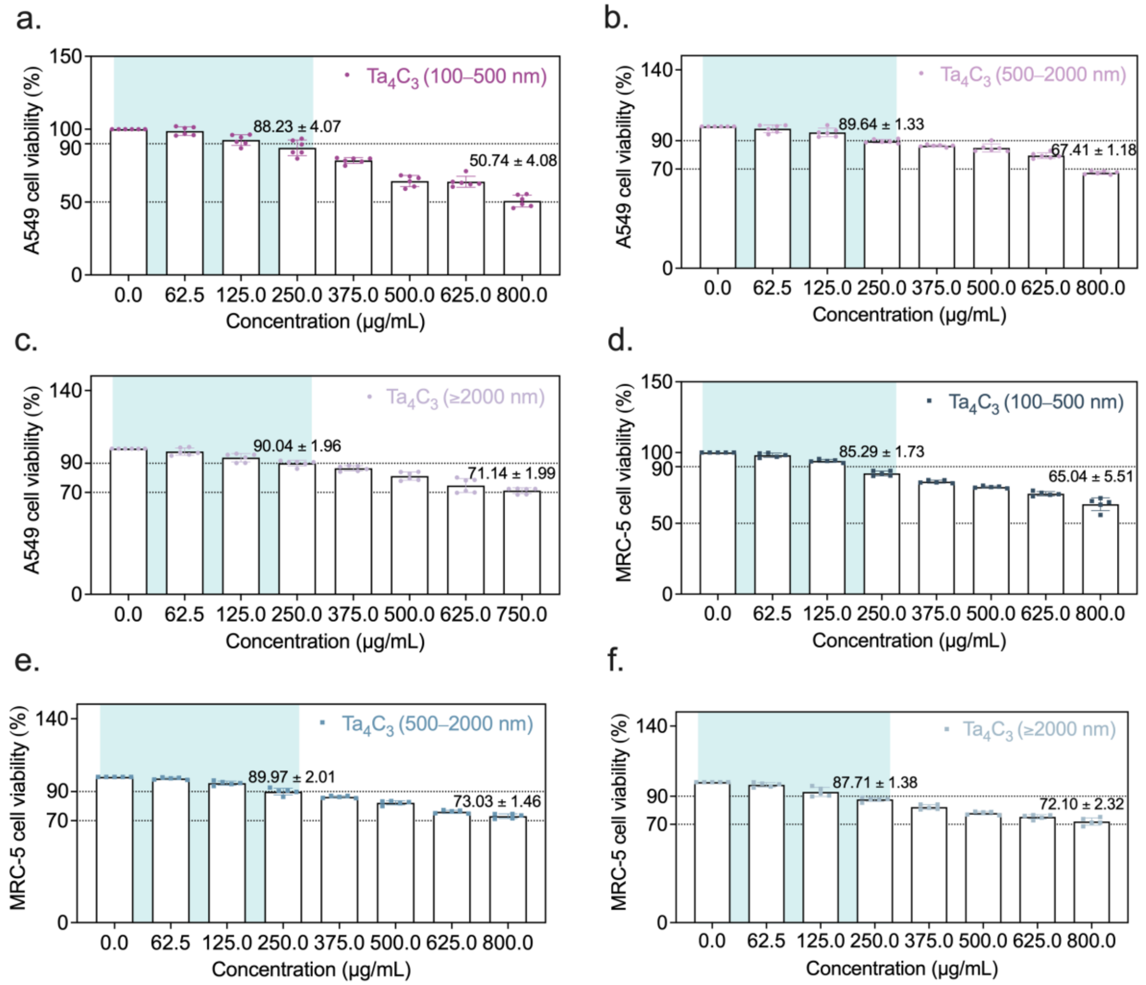

Supplementary Figure 3. Dose-dependent cytotoxicity of size-fractionated Ta_4C_3 nanosheets. Cell viability of (a–c) A549 epithelial cells and (d–f) MRC-5 fibroblasts following 48 h of exposure to increasing concentrations (0–800 μg/mL) of size-fractionated Ta_4C_3 nanosheets: (a, d) 100–500 nm, (b, e) 500–2000 nm, and (c, f) ≥2000 nm. Viability was assessed via Calcein-AM assay and normalized to untreated control groups. Data represent mean ± SD (n = 6). Horizontal dotted lines indicate the 50%, 70%, and 90% viability thresholds to facilitate comparison across cell types and sizes. The blue shaded region highlights the high-biocompatibility zone, where cell

viability remains above 90% at concentrations ranging from 0 to 250 μg/mL. These datasets serve as the basis for the dose-response curves and IC_{50} values reported in Figure 4 of the manuscript.

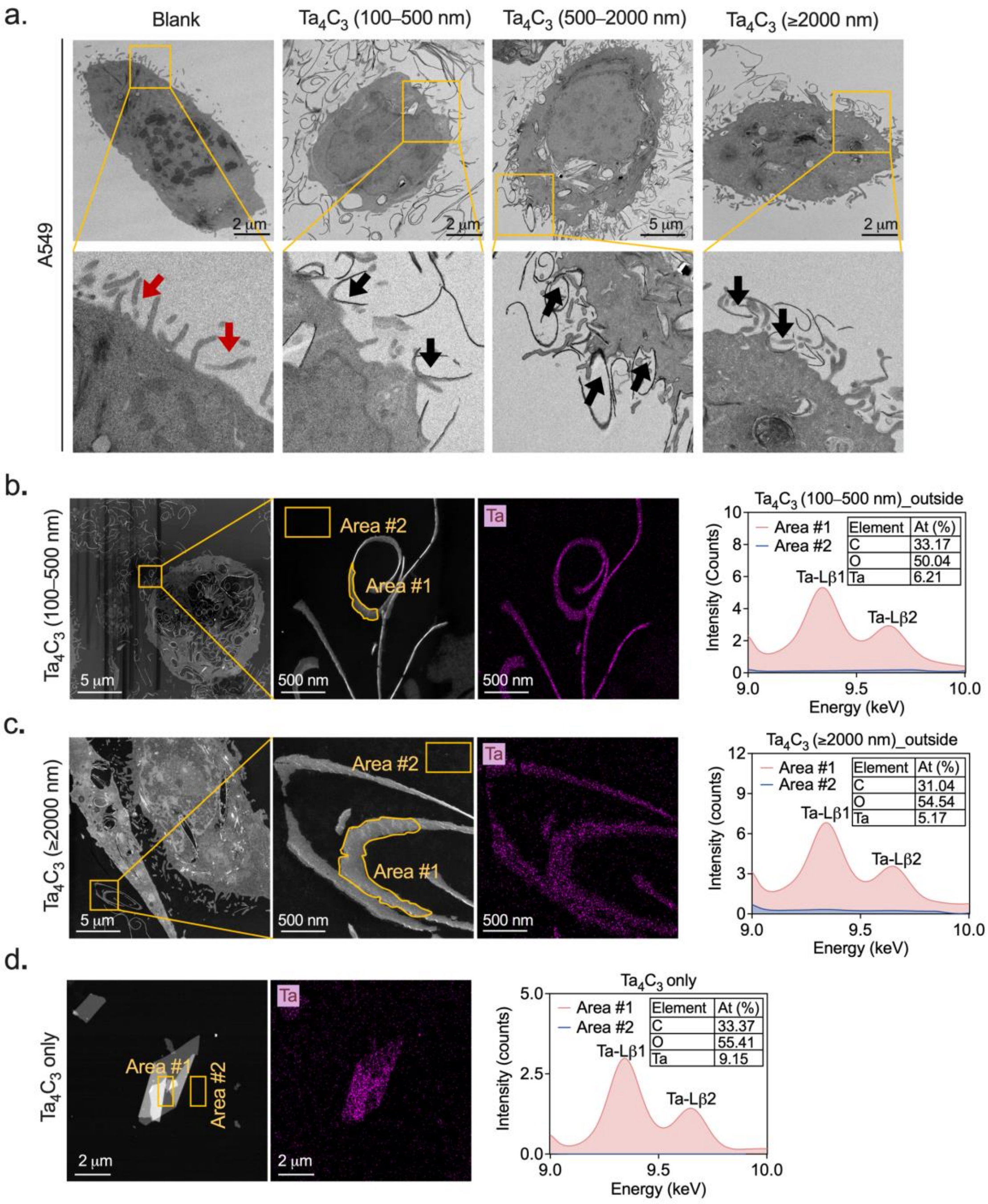

Supplementary Figure 4. Cellular internalization and elemental verification of Ta_4C_3 in A549 cells. (a) TEM micrographs illustrating the initial stages of cellular uptake in A549 cells following exposure to size-fractionated Ta_4C_3. Nanosheets exhibit a characteristic transition into strip- and circle-shaped morphologies as they adhere to and accumulate near slender cytoplasmic projections

(indicated by black arrows). (b, c) STEM-EDX elemental mapping and spectral analysis confirming the presence of Tantalum (Ta, purple) within extracellular and membrane-associated structures for (b) 100–500 nm and (c) ≥2000 nm fractions. (d) STEM-EDX reference control of pristine Ta_4C_3 nanosheets. Elemental composition and spectral intensity were determined by background subtraction (area #1-area #2), with characteristic Ta-Lβ1 and Ta-Lβ2 peaks identified. Scale bars are as indicated.

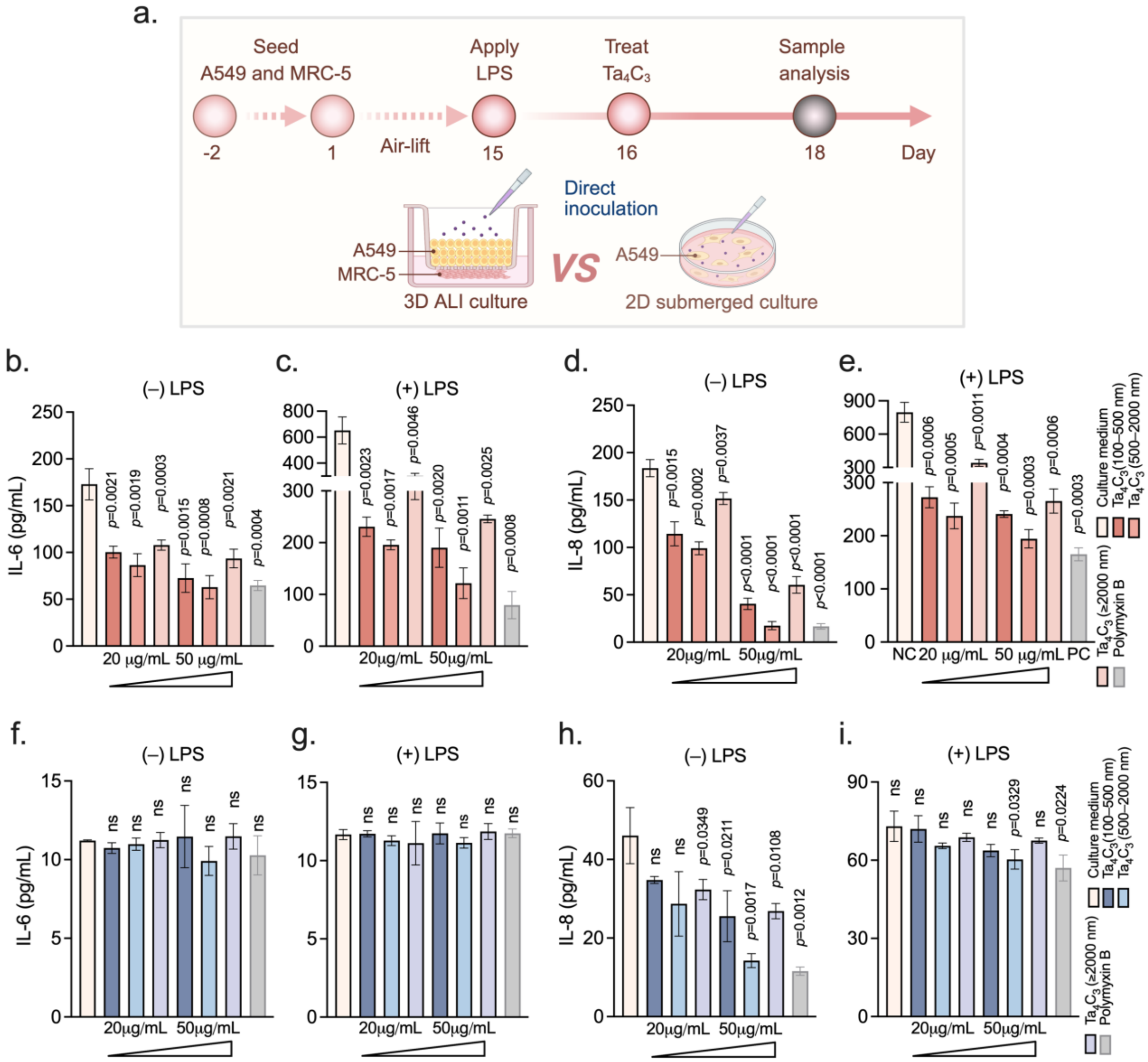

Supplementary Figure 5. Anti-inflammatory efficacy of Ta_4C_3 in 2D submerged and 3D ALI co-culture models. (a) Schematic workflow of Ta_4C_3 exposure in 2D submerged and 3D ALI co-culture (A549/MRC-5) models. (b–i) Secretion profiles of IL-6 (b, c, f, g) and IL-8 (d, e, h, i) comparing the inflammatory response across both systems. The data illustrate a dose-dependent reduction in cytokine levels at concentrations of 20 µg/mL and 50 µg /mL for all size fractions. Tissues treated only with culture medium served as the negative control. LPS-stimulated tissues without Ta_4C_3 treatment served as the positive control, while Polymyxin B-treated tissues served

as the positive therapeutic control. P values were calculated by unpaired t-test (ns = not significant). Data represent mean ± SD (n = 3 independent biological replicates).

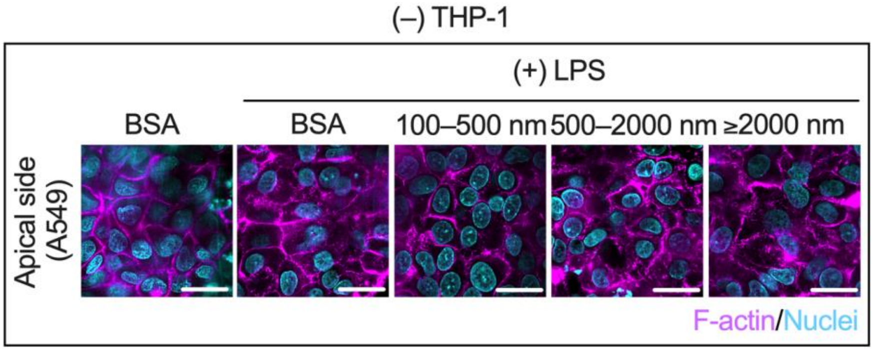

Supplementary Figure 6. Morphology changes of the 3D ALI co-culture model under pro-inflammation stimulation. Representative SDCM micrographs (apical view) of the 3D ALI co-culture model in the absense (–) of THP-1 following LPS stimulation and treatment with size-fractionated Ta_4C_3. F-actin (magenta) and nuclei (cyan) staining of the A549 layer across all treatment groups. Tissues treated only with BSA served as negative controls, while LPS-stimulated tissues without Ta_4C_3 treatment served as the positive control. Scale bars: 20 μm.

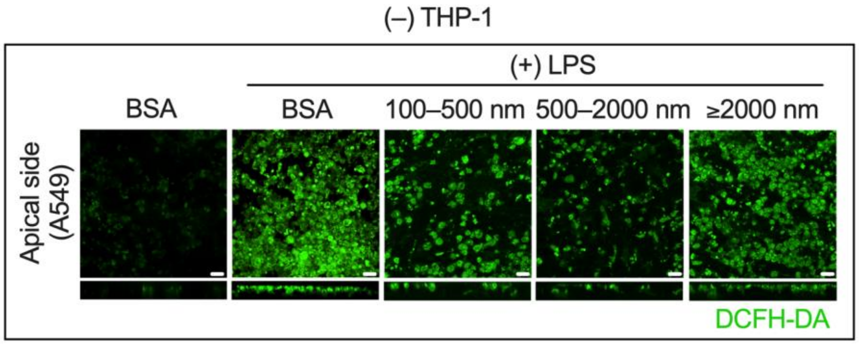

Supplementary Figure 7. Anti-oxidant efficacy of Ta_4C_3 in the 3D ALI co-culture model. Representative orthogonal SDCM micrographs of DCFH-DA staining (green) in the 3D ALI co-culture model in the absense (–) of THP-1 following LPS stimulation and treatment with size-fractionated Ta_4C_3. Tissues treated only with BSA served as negative controls. while LPS-stimulated tissues without Ta_4C_3 treatment served as the positive control. Scale bars: 20 μm.

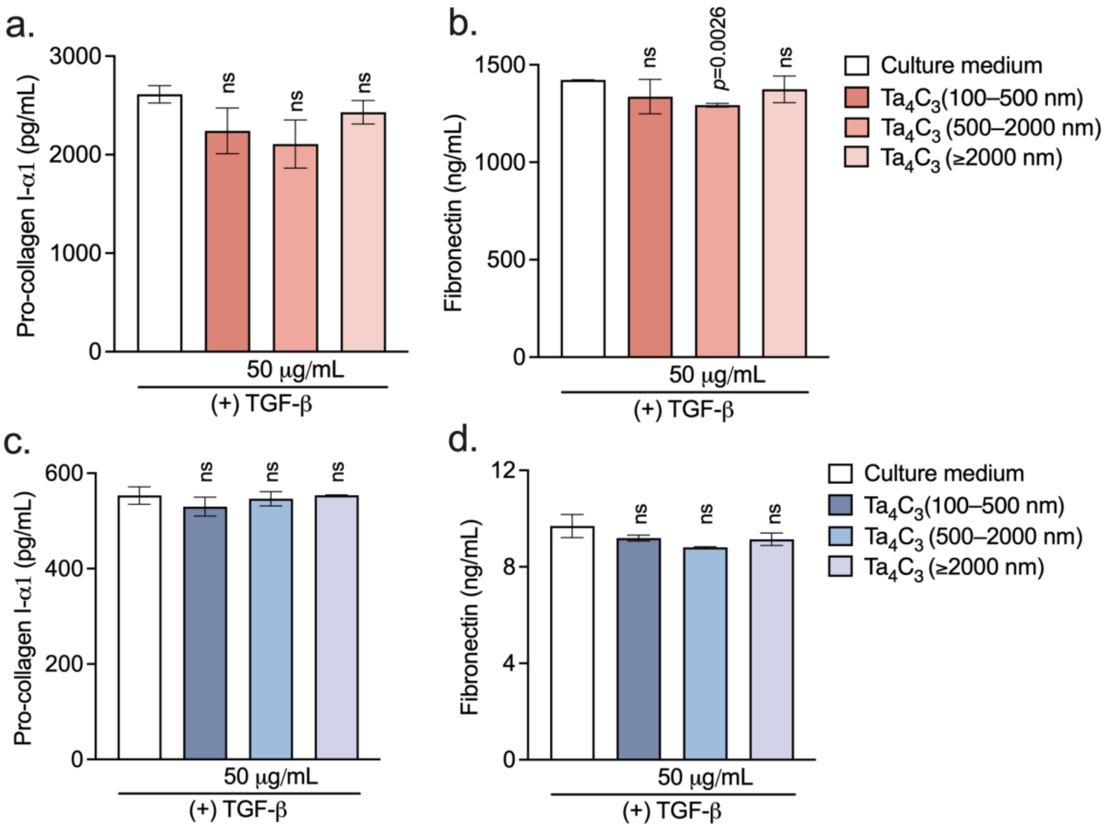

Supplementary Figure 8. Sub-therapeutic anti-fibrotic evaluation of Ta_4C_3 in 2D submerged and 3D co-culture models. Comparative secretion levels of (a, c) Pro-collagen I-α1 and (b, d) Fibronectin following TGF-β stimulation and treatment with 50 µg/mL size-fractionated Ta_4C_3. Data are shown for the (a, b) 3D ALI co-culture and (c, d) 2D submerged monoculture models. Tissues treated only with BSA served as negative controls. *P* values were calculated by unpaired *t*-test (ns = not significant). Data represent mean ± SD (n = 3 independent biological replicates).

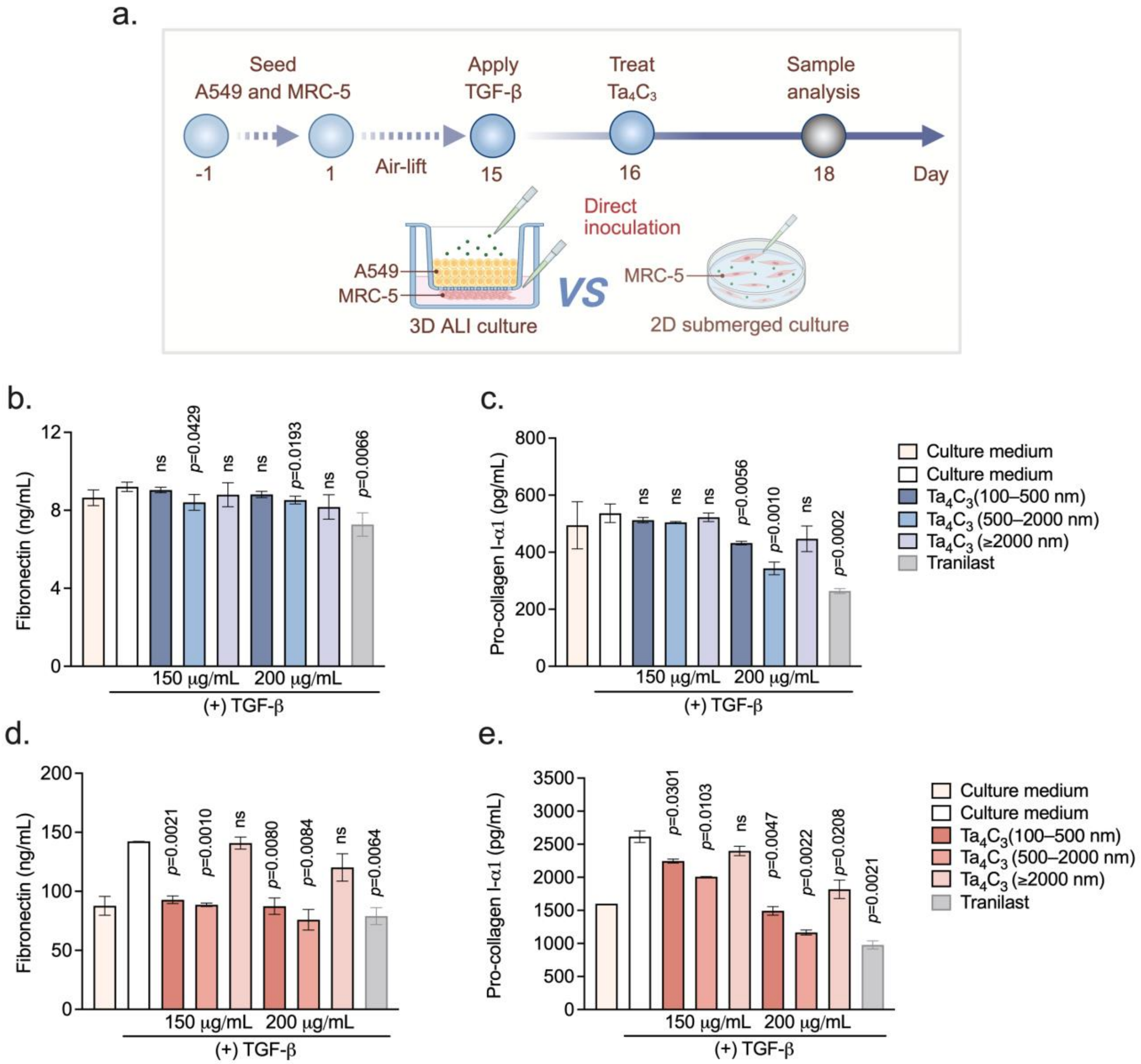

Supplementary Figure 9. Dose-dependent anti-fibrotic screening in 2D submerged and 3D co-culture models. (a) Schematic workflow of Ta_4C_3 exposure in 2D submerged and 3D ALI co-culture (A549/MRC-5) models. Comparative secretion levels of pro-fibrotic markers in (b, c) 2D submerged monoculture and (d, e) 3D ALI co-culture models following TGF-β stimulation and treatment with 150 μg/mL and 200 μg/mL size-fractionated Ta_4C_3. (b, d) Fibronectin and (c, e) Pro-collagen I-α1 release across different size fractions. Tissues treated only with culture medium served as the negative control, TGF-β -stimulated tissues without Ta_4C_3 treatment served as the

positive control, while Tranilast-treated tissues served as the positive therapeutic control. *P* values were calculated by unpaired *t*-test (ns = not significant). Data represent mean ± SD ($n = 3$, independent biological replicates).

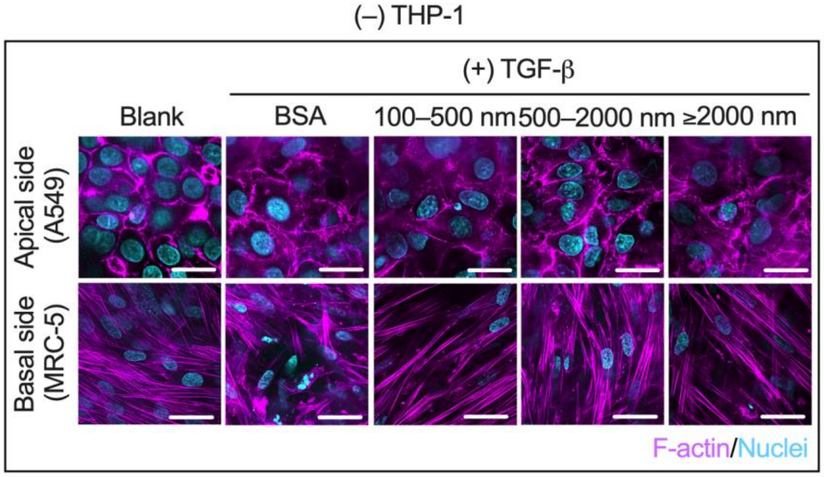

Supplementary Figure 10. Morphology changes of the 3D ALI co-culture model under pro-fibrotic stimulation. Representative SDCM micrographs (apical and basal views) of the 3D ALI co-culture model in the absence (–) of THP-1 following TGF-β stimulation and treatment with size-fractionated Ta_4C_3. F-actin (magenta) and nuclei (cyan) staining of the apical A549 layer and basal MRC-5 layer across treatment groups. Tissues treated only with BSA served as negative controls. Scale bars: 20 µm.

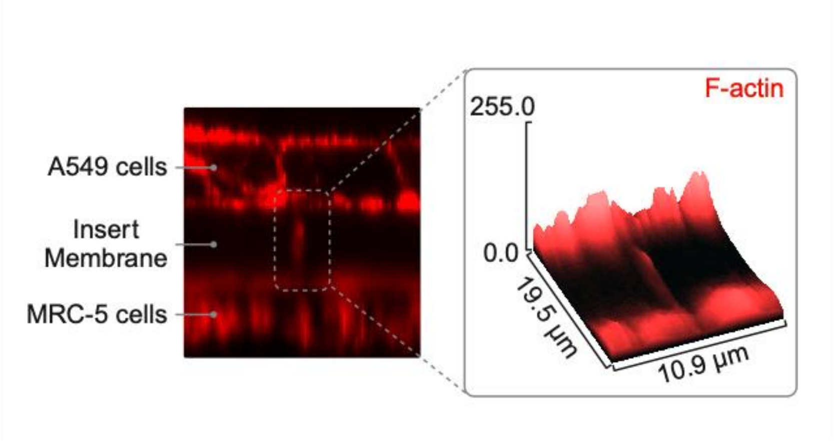

Supplementary Figure 11. Investigation of epithelial-fibroblast cross-talk. On the left shows the SDCM images of ALI multilayered co-cultures. On the right, the corresponding red fluorescence intensity profile is displayed for the highlighted region of interest, quantitatively.

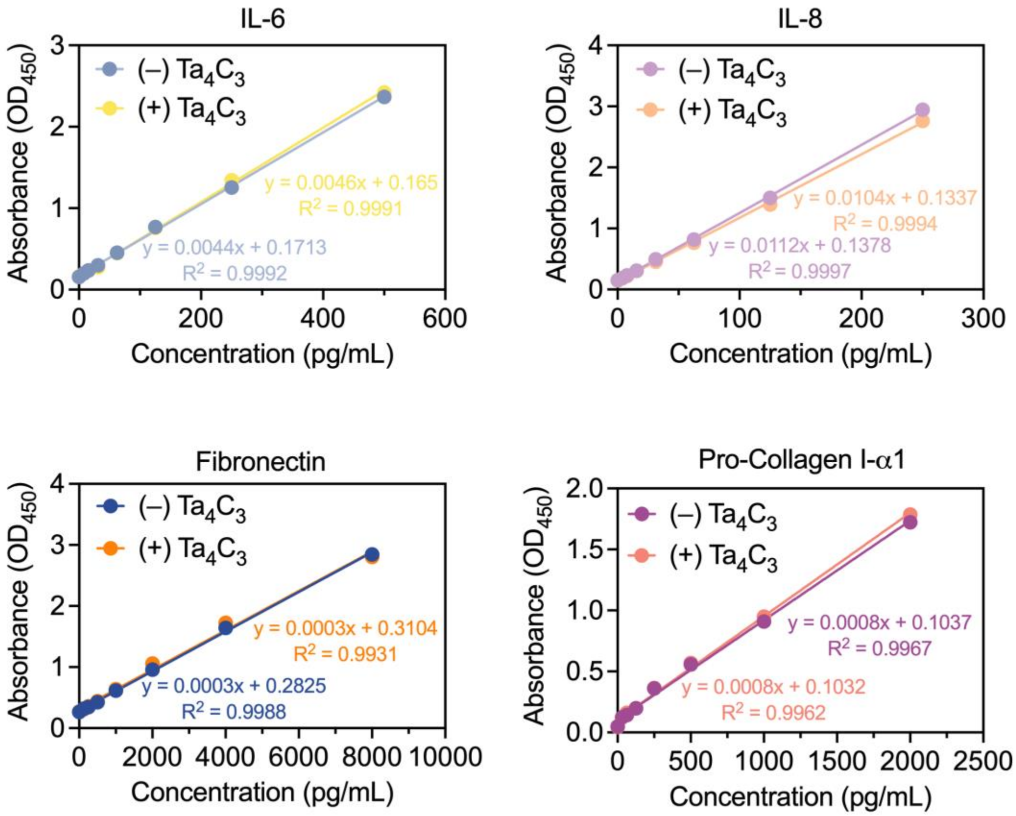

Supplementary Figure 12. To ensure that the nanosheets did not confound biomarker release by adsorbing assay reagents or quenching optical signals in ELSA assays, standard curves for proinflammatory cytokines (IL-6, IL-8) and profibrotic markers (fibronectin, pro-collagen I-α1) were generated in the absence of (–) Ta_4C_3 and presence of (+) Ta_4C_3. Absorbance values OD_{450} were plotted against standard protein concentrations.
